# Molecular typing of *Mycobacterium kansasii* using pulsed-field gel electrophoresis and a newly designed variable-number tandem repeat analysis

**DOI:** 10.1038/s41598-018-21562-z

**Published:** 2018-03-13

**Authors:** Zofia Bakuła, Anna Brzostek, Paulina Borówka, Anna Żaczek, Izabela Szulc-Kiełbik, Agata Podpora, Paweł Parniewski, Dominik Strapagiel, Jarosław Dziadek, Małgorzata Proboszcz, Jacek Bielecki, Jakko van Ingen, Tomasz Jagielski

**Affiliations:** 1 Department of Applied Microbiology, Institute of Microbiology, Faculty of Biology, University of Warsaw, Warsaw, Poland; 2 Institute of Medical Biology, Polish Academy of Sciences, Łódź, Poland; 3 Department of Anthropology, University of Łódź, Łódź, Poland; 4 Department of Biochemistry and Cell Biology, University of Rzeszów, Rzeszów, Poland; 5 Biobank Lab, Department of Molecular Biophysics, Faculty of Biology and Environmental Protection, University of Łódź, Łódź, Poland; 6 Department of Internal Medicine, Pulmonary Diseases and Allergy, Medical University of Warsaw, Warsaw, Poland; 7 Department of Medical Microbiology, Radboud University Medical Center, Nijmegen, The Netherlands

## Abstract

Molecular epidemiological studies of *Mycobacterium kansasii* are hampered by the lack of highly-discriminatory genotyping modalities. The purpose of this study was to design a new, high-resolution fingerprinting method for *M. kansasii*. Complete genome sequence of the *M. kansasii* ATCC 12478 reference strain was searched for satellite-like repetitive DNA elements comprising tandem repeats. A total of 24 variable-number tandem repeat (VNTR) loci were identified with potential discriminatory capacity. Of these, 17 were used to study polymorphism among 67 *M. kansasii* strains representing six subtypes (I-VI). The results of VNTR typing were compared with those of pulsed-field gel electrophoresis (PFGE) with AsnI digestion. Six VNTRs i.e. (VNTR 1, 2, 8, 14, 20 and 23) allow to differentiate analyzed strains with the same discriminatory capacities as use of a 17-loci panel. VNTR typing and PFGE in conjunction revealed 45 distinct patterns, including 11 clusters with 33 isolates and 34 unique patterns. The Hunter-Gaston’s discriminatory index was 0.95 and 0.66 for PFGE and VNTR typing respectively, and 0.97 for the two methods combined. In conclusion, this study delivers a new typing scheme, based on VNTR polymorphism, and recommends it as a first-line test prior to PFGE analysis in a two-step typing strategy for *M. kansasii*.

## Introduction

*Mycobacterium kansasii*, a non-tuberculous mycobacterium (NTM), is an opportunistic pathogen with a predilection for causing pulmonary disease. Since only two cases of *M. kansasii* human-to-human transmission have been documented^1,2^, the presumed source of the disease is exposure to environmental reservoirs, particularly municipal tap water^3,4^.

Although *M. kansasii* is one of the six most frequently isolated NTM species across the world^4^, little is known about the epidemiological aspects of *M. kansasii* infection including its reservoirs, contagiousness, transmission routes, and distribution in different geographical regions and among different human populations. This hampers the implementation of any prophylactic procedures and emergency therapies to control and prevent *M. kansasii* infections.

An important reason for this scanty knowledge on *M. kansasii* epidemiology is the lack of powerful tools allowing for intraspecies differentiation or typing. This, in turn, originates from a poor understanding of the genetic composition of *M. kansasii*. Until quite recently *M. kansasii* has been considered an extensively clonal species^5^. The genetic structure of *M. kansasii* has comprehensively been investigated in two late 1990s studies, which allowed the species to be classified into five distinct subtypes (I-V), based on PCR restriction enzyme analysis (PCR-REA) with the major polymorphic tandem repeat (MPTR) probe, pulsed-field gel electrophoresis (PFGE), amplified fragment length polymorphism (AFLP) analysis, and PCR-REA of the *hsp65* gene^5,6^. Somewhat later, two novel types (VI and VII) along with an intermediate I/II and atypical IIB type have been described^7,8^.

Since subtype I represented the majority of *M. kansasii* clinical isolates (42–100%) in most of the genotyping studies^5–20^, the currently available genotyping schemes do not permit differentiation between those of clinical relevance (disease-related) and those representing merely isolation, without causing a disease. However, as evidenced by Taillard *et al*., *M. kansasii* subtype I has particularly high virulence capacities in immunocompetent patients, whereas subtype II is as an opportunistic pathogen infecting immunosuppressed individuals^7^.

A number of molecular markers have been employed to explore the genetic diversity in *M. kansasii*. Whereas only slight inter-strain polymorphism has been revealed with AFLP analysis^5,10^, PFGE- and rep-PCR-based methods were more distinctive^5,6,9,13,16,19^. However, in all those studies, one-fifth (18.6%) to half of the *M. kansasii* strains (47%) were indistinguishable.

Altogether, there is currently no effective genotyping methodology for *M. kansasii*, sufficient for approaching key issues in the epidemiology of this pathogen. Hence, the formulation of a new technique, with a high discriminatory capacity is strongly required. Molecular typing methods have permitted investigation of the NTM transmission patterns^21^ discrimination between drug resistant and drug susceptible isolates^22^ and identification of isolates with high virulence capacities^23^.

Mycobacterial interspersed repetitive units (MIRUs), a specific class of variable number of tandem repeats (VNTRs), were first described as 46–101-bp DNA elements scattered at 41 loci in the chromosome of *M. tuberculosis* H37Rv^24^. Variations in the MIRU-VNTR copy number have predisposed these sequences to be used as efficient molecular markers for typing isolates of *M. tuberculosis* complex. Furthermore, since MIRU-VNTR analysis is based on PCR amplification of the respective loci, followed by determination of the sizes of the amplicons by gel electrophoresis, it is considered relatively inexpensive and easy to handle typing method. Over the last 15 years, sequences similar to MIRU-VNTRs in *M. tuberculosis*, have been identified in several NTM species, including *M. abscessus*, *M. avium*, *M. intracellulare*, *M. marinum*, and *M. ulcerans*^25–30^, and non-mycobacteria^31,32^ and used for inter-strain differentiation with a varied degree of success.

However, a multi-lous VNTR-based method has never been attempted on *M. kansasii*.

In this study, a genome of *M. kansasii* was searched for VNTR-like loci, and based on those found, a new typing scheme for *M. kansasii* was designed. The method was evaluated on a collection of 67 *M. kansasii* isolates, representatives of six subtypes (I-VI), and compared, in terms of resolution power, performance, and practicality, with PFGE analysis.

## Results

### VNTR typing

Among 1,447 VNTRs found in the genome of the *M. kansasii* ATCC 12478 reference strain (Suppl. Table 4), 24 were selected based on predefined criteria. The characteristics of those loci were described in Suppl. Table 2. Seven loci (VNTR 5, 9, 10, 12, 13, 16, 22) were excluded from the analysis due to lack of amplification (VNTR 9, 13, 22) or multiple bands (VNTR 5, 10, 12, 16, 22), as seen upon electrophoresis.

Different VNTR loci showed different ranges of allelic variability (Table 1). For three loci^14,17,24^ only two allelic variants were detected. Eight loci^3,4,7,11,15,18,21,23^ were demonstrated as three-allele types, and another two^1,6^ and three^8,19,20^ loci as four-and five-allele types, respectively. The highest level of variation was evidenced for VNTR locus 2, with seven allelic types.

**Table 1:** VNTR typing profiles of 67 *M. kansasii* isolates.

VNTR profile	VNTR locus (HGDI^a^)																	No. of isolates	Total no. of isolates (subtype)
	1 (0.22)	2 (0.32)	3 (0.33)	4 (0.34)	6 (0.29)	7 (0.33)	8 (0.38)	11 (0.29)	14 (0.09)	15 (0.14)	17 (0.32)	18 (0.33)	19 (0.34)	20 (0.38)	21 (0.34)	23 (0.2)	24 (0.19)		
a	**13**	**7**	7	4	6	6	**5**	7	5	6	5	5	11	**13**	6	3	9	39	54 (I)
b	**13**	**6**	7	4	6	6	**5**	7	5	6	5	5	11	**13**	6	3	9	5	
c	**13**	**8**	7	4	6	6	**5**	7	5	6	5	5	11	**13**	6	3	9	2	
d	**13**	**5**	7	4	6	6	**5**	7	5	6	5	5	11	**13**	6	3	9	1	
e	**13**	**7**	7	4	6	6	**7**	7	5	6	5	5	11	**13**	6	3	9	1	
f	**13**	**8**	7	4	6	6	**5**	7	5	6	5	5	11	**12**	6	3	9	1	
g	**13**	**7**	7	4	6	6	**6**	7	5	6	5	5	11	**13**	6	3	9	1	
h	**13**	**7**	7	4	6	6	**3**	7	5	6	5	5	11	**13**	6	3	9	1	
i	**13**	**7**	7	4	6	6	**5**	7	5	6	5	5	11	**12**	6	3	9	1	
j	**13**	**4**	7	4	6	6	**5**	7	5	6	5	5	11	**13**	6	3	9	1	
k	**12**	**7**	7	4	6	6	**5**	7	5	6	5	5	11	**13**	6	3	9	1	
l	**13**	**7**	**2**	**3**	1	0	3	2	**5**	6	1	0	12	12	4	**3**	3	4	7 (II)
m	**13**	**7**	**2**	**3**	1	0	3	2	**NP** ^**b**^	6	1	0	12	12	4	**3**	3	1	
n	**13**	**7**	**2**	**3**	1	0	3	2	**NP**	6	1	0	12	12	4	**0**	3	1	
o	**0**	**2**	**4**	**0**	1	0	3	2	**4**	6	1	0	12	12	4	**3**	3	1	
p	0	7	2	0	4	1	NP	7	5	MP^c^	1	1	NP	8	NP	5	9	2	2 (III)
r	MP	3	2	0	1	0	0	1	5	6	NP	0	6	NP	NP	0	9	1	1 (IV)
s	14	7	2	0	6	0	3	2	5	14	1	0	7	2	NP	MP	9	2	2 (V)
t	12	7	MP	0	5	NP	5	1	5	0	1	0	10	0	12	0	9	1	1 (VI)

Bold font indicates VNTR locus at which allelic variability within the same subtype was observed;

^a^The Hunter-Gaston’s discriminatory index;

^b^NP, no PCR product;

^c^MP, multiple-band profile.

VNTR 19 was the only locus capable of discriminating between all *M. kansasii* subtypes.

**Figure 1:**
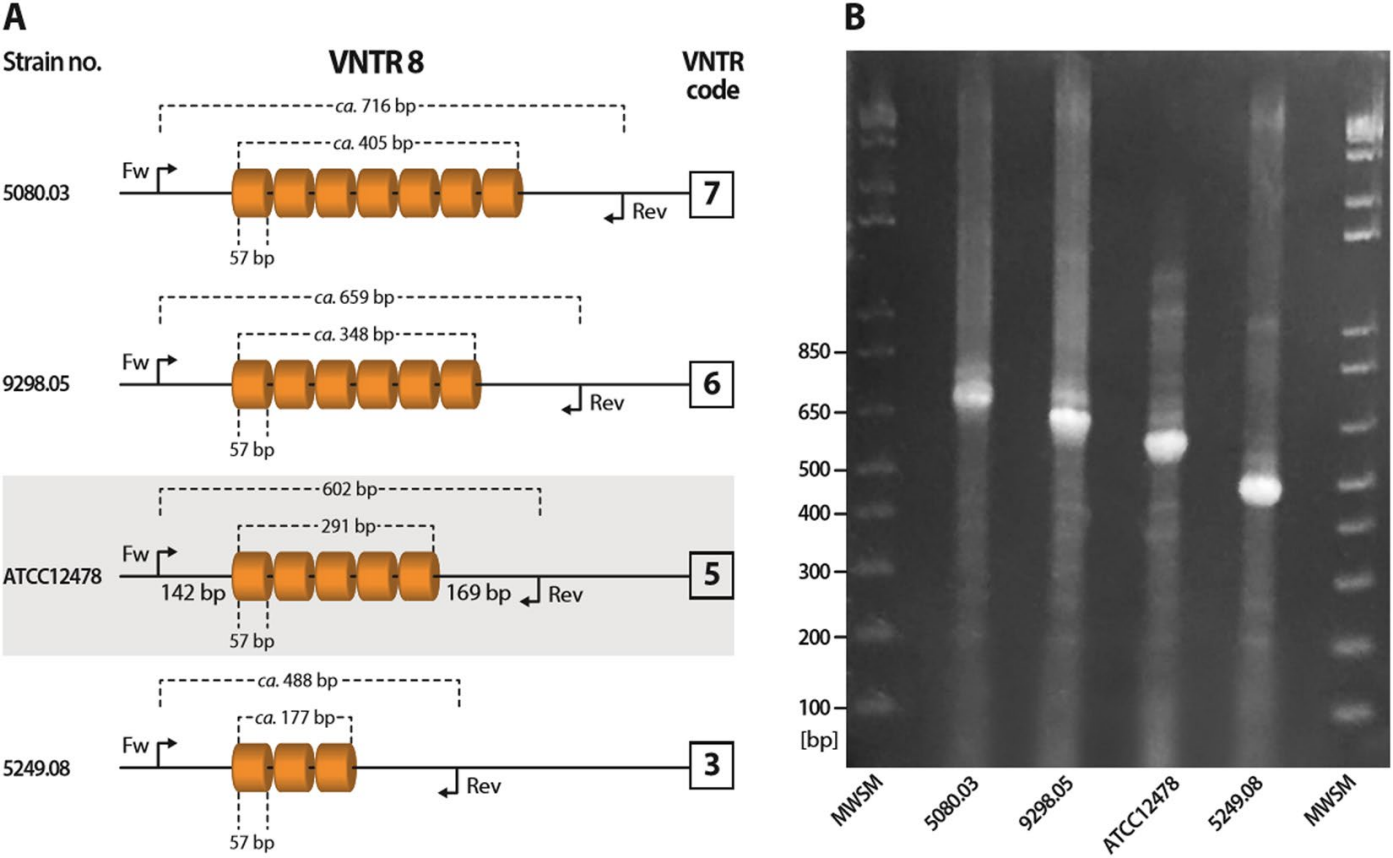
Polymorphism of the VNTR 8 locus. (**A**) Schematic representation of allelic diversity of the VNTR 8 locus among isolates under the study. (**B**) PCR-amplified VNTR 8 locus in four *M. kansasii* isolates showing bands of different length corresponding to different number of repeats within the locus. MWSM, molecular-weight size marker (100 bp DNA ladder, New England BioLabs, Ipswich, USA).

The HGDIs for each VNTR locus are shown in Table 1. The most distinguishing loci were VNTR 8 and VNTR 20 (HGDI = 0.38). Whereas the lowest diversity was observed for VNTR 14 (HGDI = 0.09). The schematic representation of allelic diversity at VNTR 8 locus is depicted on Fig. 1.

17-loci VNTR analysis produced 19 distinct profiles in total. There were six clusters with 2–39 isolates per cluster and 13 unique profiles (GDI = 0.35; HGDI = 0.66) (Table 1, Suppl. Figure 2). The clustering rate (CR) of the VNTR method was calculated at 80.6%.

**Figure 2:**
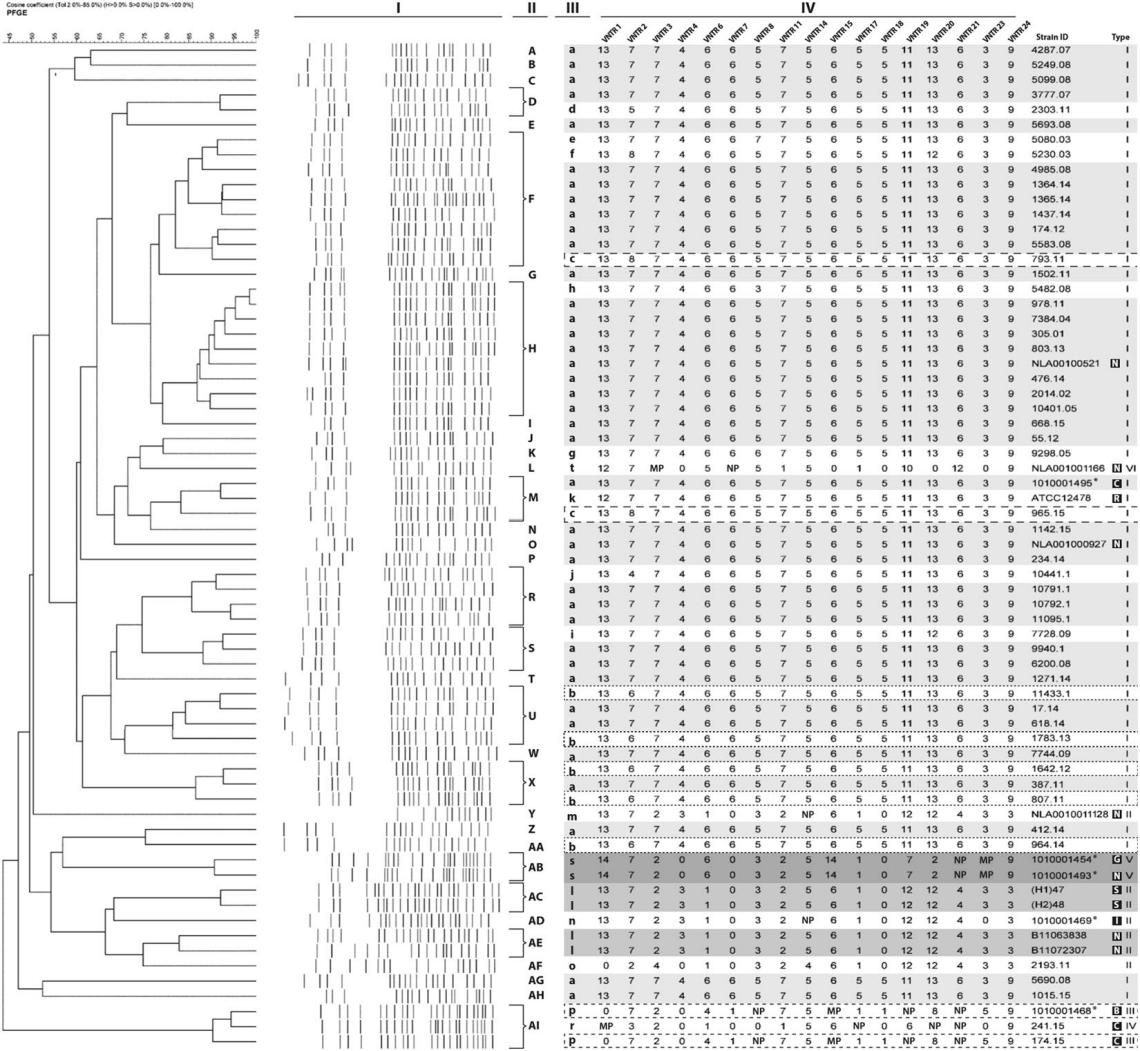
PFGE and VNTR analysis of analyzed isolates. A dendrogram constructed for 67 *M. kansasii* isolates, based on the joint results of 17-loci VNTR and PFGE profiling. I– PFGE banding patterns; II, III– Designation of PFGE (II) and VNTR profiles; IV– TR copy number at each of the 17 loci (VNTR code); VNTR codes highlighted with an identical shade of grey or boxed by dashed lines of different dash spacing correspond to different VNTR clusters; *Strains of environmental origin; Letters in black squares refer to country of strain isolation; N– the Netherlands; C– the Czech Republic; R– reference strain; G– Germany; S– Spain; I– Italy; B– Belgium; NP, no PCR product; MP, multiple-band profile. Due to large number of analysed isolates, the samples were derived from multiple experiments. The gels were processed using BioNumerics ver. 5.0 software (Applied Maths, Sint-Martens-Latem, Belgium) software in parallel.

Among 54* M. kansasii* subtype I isolates only VNTR 1, 2, 8, and 20 showed variation, generating 11 different VNTR profiles, split into 3 clusters and 8 unique profiles (GDI = 0.2; HGDI = 0.47). Cluster “a” contained almost two-thirds of the isolates (39/54; 72.2%), whereas cluster “b” and “c” included five (5/54; 9.2%) and two (2/54; 3.7%) isolates, respectively.

Six VNTRs (1–4, 14, and 23) were polymorphic among 7 *M. kansasii* subtype II isolates, and produced 4 different profiles – a four-isolate (4/7; 57.1%) cluster (“l”) and three unique profiles (GDI = 0.57, HGDI = 0.71).

Profiles “p”, “r”, “s”, and “t” were specific for *M. kansasii* type III (*n* = 2), IV (*n* = 1) V (*n* = 2), and VI (*n* = 1), respectively.

It is worth noting that the use of only six VNTRs (VNTR 1, 2, 8, 14, 20 and 23) had the same discriminatory power as the use of whole 17-loci panel (GDI = 0.35; HGDI = 0.66).

Of the six VNTR clusters, four (“a”, “l”, “p”, “s”) contained isolates from different countries, two (“a”, “p”) contained isolates of both environmental and clinical origin, and three (“a”, “b”, “l”) contained isolates of both disease-related and unrelated cases.

Loci, most difficult to assess with respect to the copy number upon gel-sizing (i.e. containing short repeats of 20 bp size or less, namely VNTR 19, 20 and 24) were sequenced to verify estimates of sequence divergence. The copy number of VNTR 19 was in full agreement with that based on gel sizing for the representatives of *M. kansasii* types I, II and VI. Yet, in the genotype V strain, a single copy difference was noted (Suppl. Table 5). For locus VNTR 20, sequencing results were obtained only for *M. kansasii* type I strain, confirming the number of copies calculated from the gel analysis of PCR products. Sequence analysis confirmed also the electrophoretically-deduced copy number at locus VNTR 24 for *M. kansasii* types I, II, and IV and VI.

### PFGE

Three restriction endonucleases, that is AseI, DraI, and XbaI were attempted in PFGE profiling. Whereas, the latter two consistently resulted in DNA band smearing, thus making the profiles unreadable (data not shown), the use of AseI provided good-quality restriction patterns with clear band separation. PFGE with AseI was performed on the entire study sample (67 isolates).

PFGE produced 33 distinct patterns, each consisting of 11–24 bands with sizes ranging between 48.5 to 485 kb. The patterns were separated into 12 clusters, each containing 2–9 isolates, and 21 unique patterns (GDI = 0.49; HGDI = 0.95) (Table 2, Suppl. Figure 1).

**Table 2:** PFGE profiles of 67 *M. kansasii* isolates.

PFGE profile	No. of isolates	Total no. of isolates (subtype)
A	1	54 (I)
B	1	
C	1	
D	2	
E	1	
F	9	
G	1	
H	9	
I	1	
J	1	
K	1	
M	3	
N	1	
O	1	
P	1	
R	4	
S	3	
T	1	
U	4	
W	1	
X	3	
Z	1	
AA	1	
AG	1	
AH	1	
Y	1	7 (II)
AC	2	
AD	1	
AE	2	
AF	1	
AI	2	2 (III)
	1	1 (IV)
AB	2	2 (V)
L	1	1 (V)

The CR of the PFGE analysis was calculated at 68.6%.

Fifty-four *M. kansasii* subtype I isolates formed 25 different profiles split into 8 clusters and 17 unique patterns (GDI = 0.46; HGDI = 0.93). Of the clusters, the most abundant were F and H, each comprising 9 isolates (9/54; 16.7%), followed by R and U, of four isolates, each (4/54; 7.4%), M, S, and X, each with three isolates (3/54; 5.6%), and D with two isolates (2/54; 3.7%).

Among seven *M. kansasii* type II isolates, there were two clusters (AC, AE), with two isolates, each (2/7; 28.6%), and three unique profiles (GDI = 0.71; HGDI = 0.9).

Type III (*n* = 2) and IV (*n* = 1) isolates shared identical PFGE pattern (AI), whereas type V (*n* = 2) and VI (*n* = 1) isolates had type-specific profiles, that is AB and L, respectively. Although some clusters were homogenous with respect to isolate’s geographical origin (e.g. F, R, U, AC, AE), source of recovery (clinical/environmental) (e.g. F, H, R, U, AB), and clinical relevance (e.g. S, AC, AE), clusters allocating isolates of different pathogenic status (F, H, R, U, X), from different countries (H, M, AI, AB) or sources (M, AI) were also observed.

### Combined analysis

Four (“a”, “b”, “c”, “l”) of the six VNTR clusters were subdivided by PFGE analysis (Table 3**;** Fig. 2). Within a major VNTR cluster “a”, comprising 39 isolates, 23 distinct pulsotypes (A-J, M-Z, AG, AH) could be distinguished. PFGE also differentiated within VNTR clusters “b” (5 isolates), “l” (4 isolates), and “c” (2 isolates), creating three (U, X, AA), two (AC, AE), and two (F, M) pulsotypes, respectively.

**Table 3:** Combined analysis of VNTR and PFGE profiling results of 67 *M. kansasii* isolates.

PFGE profile	VNTR profile	VNTR locus																	No. of isolates	Total no. of isolates (subtype)
		1	2	3	4	6	7	8	11	14	15	17	18	19	20	21	23	24		
A	a	**13**	**7**	7	4	6	6	**5**	7	5	6	5	5	11	**13**	6	3	9	1	54 (I)
B																			1	
C																			1	
D	a	**13**	**7**	7	4	6	6	**5**	7	5	6	5	5	11	**13**	6	3	9	1	
	d	**13**	**5**	7	4	6	6	**5**	7	5	6	5	5	11	**13**	6	3	9	1	
E	a	**13**	**7**	7	4	6	6	**5**	7	5	6	5	5	11	**13**	6	3	9	1	
F	a	**13**	**7**	7	4	6	6	**5**	7	5	6	5	5	11	**13**	6	3	9	6	
	c	**13**	**8**	7	4	6	6	**5**	7	5	6	5	5	11	**13**	6	3	9	1	
	e	**13**	**7**	7	4	6	6	**7**	7	5	6	5	5	11	**13**	6	3	9	1	
	f	**13**	**8**	7	4	6	6	**5**	7	5	6	5	5	11	**12**	6	3	9	1	
G	a	**13**	**7**	7	4	6	6	**5**	7	5	6	5	5	11	**13**	6	3	9	1	
H	a	**13**	**7**	7	4	6	6	**5**	7	5	6	5	5	11	**13**	6	3	9	8	
	h	**13**	**7**	7	4	6	6	**3**	7	5	6	5	5	11	**13**	6	3	9	1	
I	a	**13**	**7**	7	4	6	6	**5**	7	5	6	5	5	11	**13**	6	3	9	1	
J																			1	
K	g	**13**	**7**	7	4	6	6	**6**	7	5	6	5	5	11	**13**	6	3	9	1	
M	a	**13**	**7**	7	4	6	6	**5**	7	5	6	5	5	11	**13**	6	3	9	1	
	c	**13**	**8**	7	4	6	6	**5**	7	5	6	5	5	11	**13**	6	3	9	1	
	k	**12**	**7**	7	4	6	6	**5**	7	5	6	5	5	11	**13**	6	3	9	1	
N	a	**13**	**7**	7	4	6	6	**5**	7	5	6	5	5	11	**13**	6	3	9	1	
O																			1	
P																			1	
R	a	**13**	**7**	7	4	6	6	**5**	7	5	6	5	5	11	**13**	6	3	9	3	
	j	**13**	**4**	7	4	6	6	**5**	7	5	6	5	5	11	**13**	6	3	9	1	
S	a	**13**	**7**	7	4	6	6	**5**	7	5	6	5	5	11	**13**	6	3	9	2	
	i	**13**	**7**	7	4	6	6	**5**	7	5	6	5	5	11	**12**	6	3	9	1	
T	a	**13**	**7**	7	4	6	6	**5**	7	5	6	5	5	11	**13**	6	3	9	1	
U	a	**13**	**7**	7	4	6	6	**5**	7	5	6	5	5	11	**13**	6	3	9	2	
	b	**13**	**6**	7	4	6	6	**5**	7	5	6	5	5	11	**13**	6	3	9	2	
W	a	**13**	**7**	7	4	6	6	**5**	7	5	6	5	5	11	**13**	6	3	9	1	
X	a	**13**	**7**	7	4	6	6	**5**	7	5	6	5	5	11	**13**	6	3	9	1	
	b	**13**	**6**	7	4	6	6	**5**	7	5	6	5	5	11	**13**	6	3	9	2	
Z	a	**13**	**7**	7	4	6	6	**5**	7	5	6	5	5	11	**13**	6	3	9	1	
AA	b	**13**	**6**	7	4	6	6	**5**	7	5	6	5	5	11	**13**	6	3	9	1	
AG	a	**13**	**7**	7	4	6	6	**5**	7	5	6	5	5	11	**13**	6	3	9	1	
AH																			1	
Y	m	**13**	**7**	**2**	**3**	1	0	3	2	**NP** ^**a**^	6	1	0	12	12	4	**3**	3	1	7 (II)
AC	l	**13**	**7**	**2**	**3**	1	0	3	2	**5**	6	1	0	12	12	4	**3**	3	2	
AD	n	**13**	**7**	**2**	**3**	1	0	3	2	**NP**	6	1	0	12	12	4	**0**	3	1	
AE	l	**13**	**7**	**2**	**3**	1	0	3	2	**5**	6	1	0	12	12	4	**3**	3	2	
AF	o	**0**	**2**	**4**	**0**	1	0	3	2	**4**	6	1	0	12	12	4	**3**	3	1	
AI	p	0	7	2	0	4	1	NP	7	5	MP	1	1	NP	8	NP	5	9	2	2 (III)
	r	MP^b^	3	2	0	1	0	0	1	5	6	NP	0	6	NP	NP	0	9	1	1 (IV)
AB	s	14	7	2	0	6	0	3	2	5	14	1	0	7	2	NP	MP	9	2	2 (V)
L	t	12	7	MP	0	5	NP^b^	5	1	5	0	1	0	10	0	12	0	9	1	1 (VI)

Bold font indicates VNTR locus at which allelic variability within the same subtype was observed;

^a^NP, no PCR product;

^b^MP, multiple-band profile.

Contrariwise, of 12 PFGE clusters, nine (D, F, H, M, R, S, U, X, AI) were further separated upon VNTR typing (Table 3). Two major pulsotype clusters (F and H), of nine isolates each, could be subdivided into four (“a”, “c”, “e”, “f”) and two (“a”, “h”) VNTR clusters, respectively. Clusters R and U, each comprising four isolates, were both split into two VNTR clusters (“a”, “j” and “a”, “b”). A three-isolate cluster M and a two-isolate cluster D were split into three (“a”, “c”, “k”), and two (“a”, “d”) VNTR clusters, accordingly. Patterns S, X, and AI, each with three isolates, were subdivided into two different VNTR clusters (“a”, “i”; “m”, “b”; “p”, “r”).

A combined VNTR-PFGE analysis resolved 45 distinct patterns, separated into 11 clusters totaling 33 isolates and 34 unique patterns (GDI = 0.67; HGDI = 0.97). The CR of the combined analysis was 49.2%.

*M. kansasii* subtype I isolates were divided into 36 different profiles or seven clusters, 2–8 isolates, each and 29 unique profiles (GDI = 0.67; HGDI = 0.96). The largest clusters (H/a; F/a) comprised eight (8/67; 11.9%) and six isolates (6/67; 8.9%), respectively.

Subtype II isolates either belonged to one of two clusters (l/AC; l/AE), each of two isolates or harbored unique profiles (GDI = 0.71; HGDI = 0.9).

Isolates of subtype III (*n* = 2), IV (*n* = 1), V (*n* = 1), and VI (*n* = 1) had their type-specific merged profiles.

Of the eleven VNTR-PFGE clusters, three (a/H; p/AI; s/AB) contained isolates from more than one country and three (a/F; a/H; b/X) contained both clinically relevant and irrelevant isolates. One cluster (p/AI) accommodated isolates of both clinical and environmental origin.

## Discussion

In the epidemiology of infectious diseases, including those of mycobacterial etiology, the key issue is disclosing sources of infection, transmission links, and dissemination in the environment. For this to be accomplished, high resolution inter-strain discrimination or typing is of utmost importance^33^.

Molecular typing methods have substantially improved our knowledge of the epidemiology of mycobacterial infections and genuinely assisted in the fight against them.

A significant source of the genetic polymorphism in mycobacteria, and one of the main driving forces of their genome evolution, are repetitive DNA elements. One such large group of repeated DNA are insertion sequences (ISs)^5,33–35^. Equally important, as ISs, group of repetitive DNA elements are tandem repeats (TRs) alias variable number of tandem repeats (VNTRs), which are short direct repeats organized as head-to-tail arrays, classified as micro-, mini- or macrosatellites, depending on their repeat unit size^36^. The first VNTRs described in mycobacteria were those identified in *M. tuberculosis*, collectively referred to as mycobacterial interspersed repetitive units (MIRUs)^37^. Although the panel of MIRU-VNTR loci has been modified over the years, the standardized 24-locus MIRU-VNTR typing is currently recognized as the reference typing system for *M. tuberculosis* complex^38–40^. The principle of the VNTR-based typing modalities involves PCR amplification of a predefined set of VNTR loci, by using primers annealing to their flanking sequences, and amplicon size determination to deduce the number of TR units within each locus. At the end, a numerical code is developed, which corresponds to the numbers of repeats at each locus and serves as a strain’s molecular fingerprint. All this renders multiple-locus VNTR analysis (MLVA) robust, easy-to-perform, time- and cost-effective. The portability of the results (MLVA codes) facilitates their storage and exchange between laboratories. More importantly, the discriminatory power of MLVA is often high, surpassing that of other typing schemes^41,42^.

VNTRs have also been described in several NTM species, including *M. abscessus*, *M. avium, M. intracellulare*, and *M. marinum*^25,27,29,43–46^. However, until this study, the only evidence for the existence of VNTR-like loci in *M. kansasii* came from a 30-year-old study by Hermans *et al*.^47^. DNA homologous to a major polymorphic tandem repeat (MPTR), originating from *M. tuberculosis*, was shown to be present in *M. kansasii* and *M. gordonae*^47^. In this work, the VNTRs were searched directly in the *M. kansasii* genomes. Based on the applied criteria, a total of 24 VNTR loci were identified in the genome of the *M. kansasii* ATCC 12478 reference strain. Only 17 of the VNTR loci were applicable as genetic markers in this species fingerprinting. Furthermore, the use of only six VNTRs (VNTR 1, 2, 8, 14, 20 and 23) showed identical strain polymorphism as the use of a whole 17-loci panel. The advantage of using this smaller panel of VNTRs is a reduction in time and cost of the analysis by almost three-fold.

VNTR locus 19 was efficient in differentiating between all *M. kansasii* types except type III, whose isolates failed to produce a PCR product. Nevertheless, given the rarity of type III, PCR amplification of VNTR locus 19 offers an alternative to more lengthy, standard PCR-REA assays, for discrimination of *M. kansasii* genotypes, including two most prevalent, clinical types I and II.

The reason for the detected difference in the VNTR 19 copy number for *M. kansasii* type V deduced from gel sizing (i.e. 8 copies) and direct sequencing (i.e. 7 copies) may lie in the miscalculation of the band size on the gel. Inaccurate allele calling is one of the major nontechnical error affecting the reproducibility of the VNTR typing^48^.

The overall discriminatory power of the 17-locus typing assay was calculated at 0.66. This was somewhat lower than what was reported for VNTR typing schemes in other NTM, including *M. abscessus* (13–18 loci, HGDI = 0.96–0.98)^29,49,50^, *M. avium* (5–8 loci, HGDI from 0.74 to 0.95)^44,51–53^ or *M. intracellulare* (15–16 loci, 0.98–0.99)^27,45,54,55^. At the same time, the genetic diversity of the population studied, measured by the number of different VNTR profiles identified per study sample, was found to be rather moderate when compared to other VNTR-typed NTM (GDI = 0.35 *vs* 0.1–0.84)^25,27,29,44,54–56^.

As far as the evolutionary genomics is concerned, mycobacteria are remarkably homogeneous at the molecular level. This is also reflected by a slow molecular clock of VNTR loci. For instance, in *M. tuberculosis*, a mean mutation rate per VNTR locus per year was estimated at 10^−4^ which translates in a mean of 0.05 pairwise (single-locus) changes per 24-locus genotype expected in sets of isolates originated from a same clone over 10 years^57^. The low discriminatory power of the 17-locus set for *M. kansasii* compared to other NTM may indicate that the rate of VNTR variation in *M. kansasii* is exceptionally low.

The clustering rate, often used as a proxy for transmission or an outbreak, exceeded, with our method, 80%. This was higher than the analogous rate for *M. abscessus* (34.2–48.2%)^29,49^ or *M. intracellulare* (31–55%)^27,54,56^ and lower than for *M. avium* (95.6–95.9%)^25,44^. Given a long collection time of the study sample (16 years) and the fact that the isolates were of different origins (both environmental and clinical), and from different geographical locales, the overall high CR reflects genome homoplasy, which is a product of convergent evolution, rather than ongoing active transmission.

Interestingly, isolate 2193.11 had VNTR pattern somewhat different than other subtype II isolates in loci 1, 2, 3 and 4. Its unique VNTR profile might be associated with geographical origin (this isolate was the only subtype II isolate from Poland).

Since the mid-1990s, PFGE has been the mainstay for molecular typing of NTM. Although, a number of methods have been introduced, over these two decades, PFGE still holds the leading position as a typing scheme in the molecular epidemiological studies of NTM diseases, including those due to *M. kansasii*^33,58^. This is because PFGE outrivals most of the later-invented typing methods in terms of discriminatory potential^5,59^.

The results from this study shows PFGE comparably discriminatory as in a study by Zhang *et al*.^13^. By performing three PFGE assays, with different enzymes (XbaI, DraI, and AseI) the GDI was similar between the assays and much the same as in our analysis (0.39–0.49 *vs* 0.49)^13^. Much lower GDI values for *M. kansasii* PFGE typing were reported by other authors, even when using the same enzymes, AseI (GDI = 0.25) or DraI (0.04–0.23) and SpeI (GDI = 0.18)^5–7,19^.

As for the clustering, our results were again close to those of Zhang *et al*.^13^ (CR = 68% *vs* 61–69%), albeit rather distant to those from the remaining four aforesaid studies, with the clustering rate ranging from 88.3 to 99.3%^5–7,19^. These differences may be explained by geographical- and/or population-related specificities of the study samples. Also, some technicalities might be at play. Since PFGE depends chiefly on DNA quality, the typing results can be influenced by a method of DNA isolation, electrophoresis/running conditions, and the puslotype interpretative criteria applied (e.g. correlation algorithms, cutoff values). Whereas PFGE is the most powerful typing system for *M. kansasii*, it is time-consuming, labor-intensive, and resource- and expertise-demanding. Thus, a new, simple, cost-effective and high-throughput typing method would be of great advantage. All these criteria are met by VNTR typing, designed in this study. Yet, the discriminatory ability of MLVA was lower compared to PFGE analysis. This was apparent both from the GDI (0.35 *vs* 0.49) and HGDI (0.66 *vs* 0.95) scores.

Encouragingly, when the two methods were used together, the resolution power of such combination increased over that of PFGE alone, as reflected by both diversity and discriminatory indexes (GDI = 0.49 *vs* 0.67; HGDI = 0.95 *vs* 0.97). At the same time, the clustering rate noticeably decreased by 31% and 19% when compared with MLVA and PFGE alone, respectively (49% *vs* 68% and 80%). These observations prompted us to propose a two-step typing strategy for *M. kansasii*, which involves MLVA as a first screening method, performed on the entire study sample, followed by PFGE profiling, performed only within the VNTR-defined clusters.

In conclusion, this study delivers a new typing scheme, based on VNTR polymorphisms, and recommends it as a first-line test prior to PFGE analysis in a two-step typing strategy for *M. kansasii*. This strategy, though requiring evaluation against large-scale samples, offers a promising tool for mapping outbreaks and delineating transmission patterns of *M. kansasii* infections.

## Methods

### Isolates

A total of 67 *M. kansasii* isolates, representing six of the species subtypes (I-VI) were included in the study (Suppl. Table 1). The isolates were purchased from the American Type Culture Collection (ATCC) (*n* = 1) or collected over a period of 2000–2015 from Poland (*n* = 51), the Netherlands (*n* = 7), the Czech Republic (*n* = 3), Spain (*n* = 2), Belgium (*n* = 1), Germany (*n* = 1) and Italy (*n* = 1). Sixty-two isolates were of clinical origin and represented as many unrelated patients diagnosed as having (or not) *M. kansasii* disease, according to the criteria of the American Thoracic Society (ATS)^3^. Five isolates were recovered from different environmental sites (Suppl. Table 1).

The isolates were identified as *M. kansasii* by using high pressure liquid chromatography (HPLC) methodology, in accordance with the Centers for Disease Control and Prevention (CDC) guidelines^60^ or by means of the GenoType Mycobacterium CM/AS assay (Hain Lifescience, Nehren, Germany).

All isolates were cultured on Löwenstein-Jensen (L-J) medium. Genomic DNA was extracted with the AMPLICOR Respiratory Specimen Preparation Kit (Roche, Basel, Switzerland). Subtyping of *M. kansasii* isolates was performed upon PCR-REA analysis for the *hsp65*, *rpoB*, and *tuf* genes, as previously described^61–63^.

**PFGE**. The PFGE analysis was performed as described previously by Kwenda *et al*.^19^ with modifications (Suppl. Materials and Methods).

The gel images were analyzed using BioNumerics ver. 5.0 software (Applied Maths, Sint-Martens-Latem, Belgium). Three molecular-weight size marker (MWSM) lanes in each 15-well gel enabled normalization within and across gels.

Cosine correlation algorithm was used to define PFGE profiles. Band positions were assigned manually, with computer assistance, and the band tolerance was set at 2%. Two isolates exhibiting >80.4% profile similarity were considered clonal. This cut-off value was derived empirically from an analysis of a number of PFGE profiles of the same isolate, in two independent PFGE assays.

### Search for VNTR loci and VNTR typing

The whole-genome sequence of the ATCC 12478 *M. kansasii* reference strain (GenBank, NCBI, Reference Sequence: NC_022663.1) was screened for repetitive DNA elements with the Tandem Repeats Finder Version 4.00^64^ and visualized by the Vector NTI Software (Thermo Fisher Scientific, Waltham, USA). The results were filtered on the basis of following criteria: (i) minimum and maximum fragment size obtained on agarose gel: 100 bp and 2,000 bp, respectively (ii) minimum number of repeat units: 4.5. Twenty-four loci were then selected on the basis of 93% of conservation between the VNTRs.

The flanking sequences, of each VNTR locus, determined in the *M. kansasii* ATCC 12478 reference strain, and several other *M. kansasii* clinical strains^65,66^ with the CLC Genomics Workbench Software 8 (Qiagen, Nehren, Germany), were used to design oligonucleotide primers and PCR protocols with the

Vector NTI Software (Thermo Fisher Scientific, Waltham, USA) (Suppl. Table 2).

The PCR reactions were performed with a TopTaq Master Mix kit, as recommended by the manufacturer (Qiagen, Hilden, Germany) with 50 ng of template DNA in a final volume of 25 µL. After initial denaturation at 95 °C for 3 min, the reaction mixture was run through 35 cycles of denaturation at 95 °C for 30 s, annealing at a temperature specific for particular VNTR loci (Suppl. Table 2) for 30 s, and extension at 72 °C for 60 s, followed by a final extension at 72 °C for 5 min. The amplicons were separated electrophoretically at 3.5 V/cm in 1% agarose gels in 0.5× TBE buffer and visualized by staining with ethidium bromide (0.5 μg/mL) and exposure to UV light (λ = 320 nm).

Seven (i.e. VNTR 5, 9, 10, 12, 13, 16, 22) of the 24 selected loci were excluded from further analysis due to lack of amplification^9,13,22^ or multiple bands^5,10,12,16,22^, as seen upon electrophoresis.

To assign the number of alleles, at each VNTR locus, corresponding to the amplicon sizes, the amplicon-length-based allele calling table was used (Suppl. Table 3). The table was configured following the computer-assisted analysis of the VNTR sequences of the *M. kansasii* ATCC 12478 reference strain. Shortly, the *in silico*-deduced number of repeats, at each locus, was rounded to the closest integer value. The number of repeats, below or above this value were assigned to amplicons’ lengths, calculated by adding or subtracting a multiple of the repeat size, at a given locus, from the amplicon size, determined for the *M. kansasii* ATCC 12478 reference strain.

For each isolate, the final result was a 17-digit VNTR code, corresponding to the number of repeats at each VNTR locus.

The VNTR copy number for loci containing 20 bp or less (i.e. VNTR 19, 20, 24) was verified for *M. kansasii* subtypes representatives by using Sanger technology (Suppl. Table 5).

### Cluster definition

A PFGE cluster was defined as two or more isolates assumed as clonal, based on the 80.4% cutoff value of the similarity between two PFGE patterns. A VNTR cluster was defined as two or more isolates sharing identical 17-loci VNTR typing profiles.

A PFGE-VNTR combined cluster was defined as two or more isolates sharing at least 80.4% cutoff value of the similarity between two PFGE patterns and identical 17-loci VNTR typing profiles.

Clustering rate was defined as percentage of clustered isolates among of all isolates genotyped^67^.

### Construction of dendrograms

Similarities were calculated by the Cosine coefficient (PFGE and PFGE and VNTR typing combined) or Pearson’s correlation coefficient (VNTR typing) algorithms. Dendrograms were constructed with BioNumerics ver. 5.0 software (Applied Maths, Sint-Martens-Latem, Belgium) by using the unweighted pair-group method with arithmetic averages (UPGMA) with 2% band position tolerance.

### Calculation of discriminatory power

As a numerical index for the discriminatory power of each typing method, genetic diversity index (GDI) and Hunter and Gaston discriminatory index (HGDI) were used.

GDI was calculated, for each typing method, as a quotient of the total number of genetic patterns to the total number of isolates. The HGDI was calculated with the following formula:

$$DI=1-[\frac{1}{N(N-1)}]{\sum }^{}nj(nj-1),$$

where *N* is the total number of isolates, and *nj* is the number of isolates representing each type^68^.

## Electronic supplementary material

Supplementary Material
