# Supplementary material for: Molecular typing of Mycobacterium kansasii using pulsed-field gel electrophoresis and a newly designed variable-number tandem repeat analysis
Source: Sci Rep. 2018 Mar 13;8:4462. doi: 10.1038/s41598-018-21562-z (PMC5849605; doi:10.1038/s41598-018-21562-z)
Supplement: Supplementary file 1 — Supplementary Material [file 41598_2018_21562_MOESM1_ESM.docx]

**SUPPLEMENTARY MATERIAL**

**Molecular typing of *Mycobacterium kansasii* using pulsed-field gel electrophoresis and a newly designed variable-number tandem repeat analysis**

Zofia Bakuła^1^, Anna Brzostek^2^, Paulina Borówka^3^, Anna Żaczek^4^, Izabela Szulc-Kiełbik^2^, Agata Podpora^1^, Paweł Parniewski^2^, Dominik Strapagiel^5^, Jarosław Dziadek^2^, Małgorzata Proboszcz^6^, Jacek Bielecki^1^, Jakko van Ingen^7^, Tomasz Jagielski^1*^

^1^Department of Applied Microbiology, Institute of Microbiology, Faculty of Biology, University of Warsaw, Warsaw, Poland

^2^Institute of Medical Biology, Polish Academy of Sciences, Łódź, Poland

^3^Department of Anthropology, University of Łódź, Łódź, Poland

^4^Department of Biochemistry and Cell Biology, University of Rzeszów, Rzeszów, Poland

^5^Biobank Lab, Department of Molecular Biophysics, Faculty of Biology and Environmental Protection, University of Łódź, Łódź, Poland

^6^Department of Internal Medicine, Pulmonary Diseases and Allergy, Medical University of Warsaw, Warsaw, Poland

^7^Department of Medical Microbiology, Radboud University Medical Center, Nijmegen, The Netherlands

***Corresponding author:** Tomasz Jagielski, PhD, Department of Applied Microbiology, Institute of Microbiology, Faculty of Biology, University of Warsaw, I. Miecznikowa 1, 02-096 Warsaw. Phone: +48 (0) 22 55 41 431; Fax: +48 (0) 22 55 41 402, E-mail: [t.jagielski@biol.uw.edu.pl](mailto:t.jagielski@biol.uw.edu.pl)

**SUPPLEMENTARY MATERIAL AND METHODS**

**PFGE.** Bacterial cells were grown in 10 mL of Middlebrook 7H9 broth supplemented with OADC and glycerol, as recommended by manufacturer (Becton Dickinson, Franklin Lakes, USA) at 37ºC with shaking (150 rpm) until turbidity at OD_550nm_ reached 2.0. The pellets from 3-mL cultures, collected by centrifugation at 3000 rpm for 10 min, were washed in 500 µL of TE buffer (pH=8.0), and resuspended in 200 µL of TE buffer (pH=8.0). Then, 200 µL of prewarmed to 55ºC 2% low melting point agarose (Bio-Rad Laboratories, Hercules, USA) was added, and the mixture was poured into plug molds (Bio-Rad Laboratories, Hercules, USA). The plugs were then solidified for 30 min at RT, followed by incubation in TE buffer (pH=8.0) with 4 mg/mL of lysozyme (Sigma-Aldrich, Steinheim, Germany) for 48 hours at 37ºC, with the enzyme refreshed post 24 hours. Next, the plugs were incubated in ESP solution (2 mg/mL of proteinase K (Sigma-Aldrich, Steinheim, Germany) and 1% N-lauroylsarcosine in 0.5 EDTA, pH=8.0) for seven days at 55ºC, with the enzyme refreshed at the fifth day. After incubation, the plugs were cut into 3-mm slices, and washed four times in TE buffer (pH=8.0), each time for an hour. After equilibration, with a restriction buffer (Thermo Scientific, Waltham, USA) for one hour at RT, the agarose-embedded DNA was digested overnight with 30U of AseI, DraI, or XbaI enzyme (Thermo Scientific, Waltham, USA). Electrophoresis was performed with CHEF Mapper® XA System (Bio-Rad Laboratories, Hercules, USA) using 2% pulsed field gel electrophoresis agarose (Bio-Rad Laboratories, Hercules, USA) in 0.5x TBE buffer. Gel was pre-equilibrated for one hour at 14ºC and then run 48 hours at 14ºC, under the following conditions: angle, 120º; gradient, 6V/cm; pump setting, 70 pmb; ramp, linear; and switch times: 5s-35s. As a molecular-weight size marker (MWSM), Lambda PFG Ladder (New England Biolabs, Ipswich, USA) of the 48.5-727.5-kb size range was used. Gels were stained with ethidium bromide (0.5 µg/mL), rinsed with distilled water, and imaged on BioDoc-It® Imaging Systems (UVP, Upland, USA).

**SUPPLEMENTARY FIGURES**

**SUPPLEMENTARY FIGURE 1. PFGE typing.** A dendrogram for cluster analysis was based on DNA restriction banding patterns of the *M. kansasii* isolates using AseI enzyme and constructed according to the Cosine correlation algorithm. Due to large number of analysed isolates, the samples were derived from multiple experiments. The gels were processed using BioNumerics ver. 5.0 software (Applied Maths, Sint-Martens-Latem, Belgium) software in parallel.


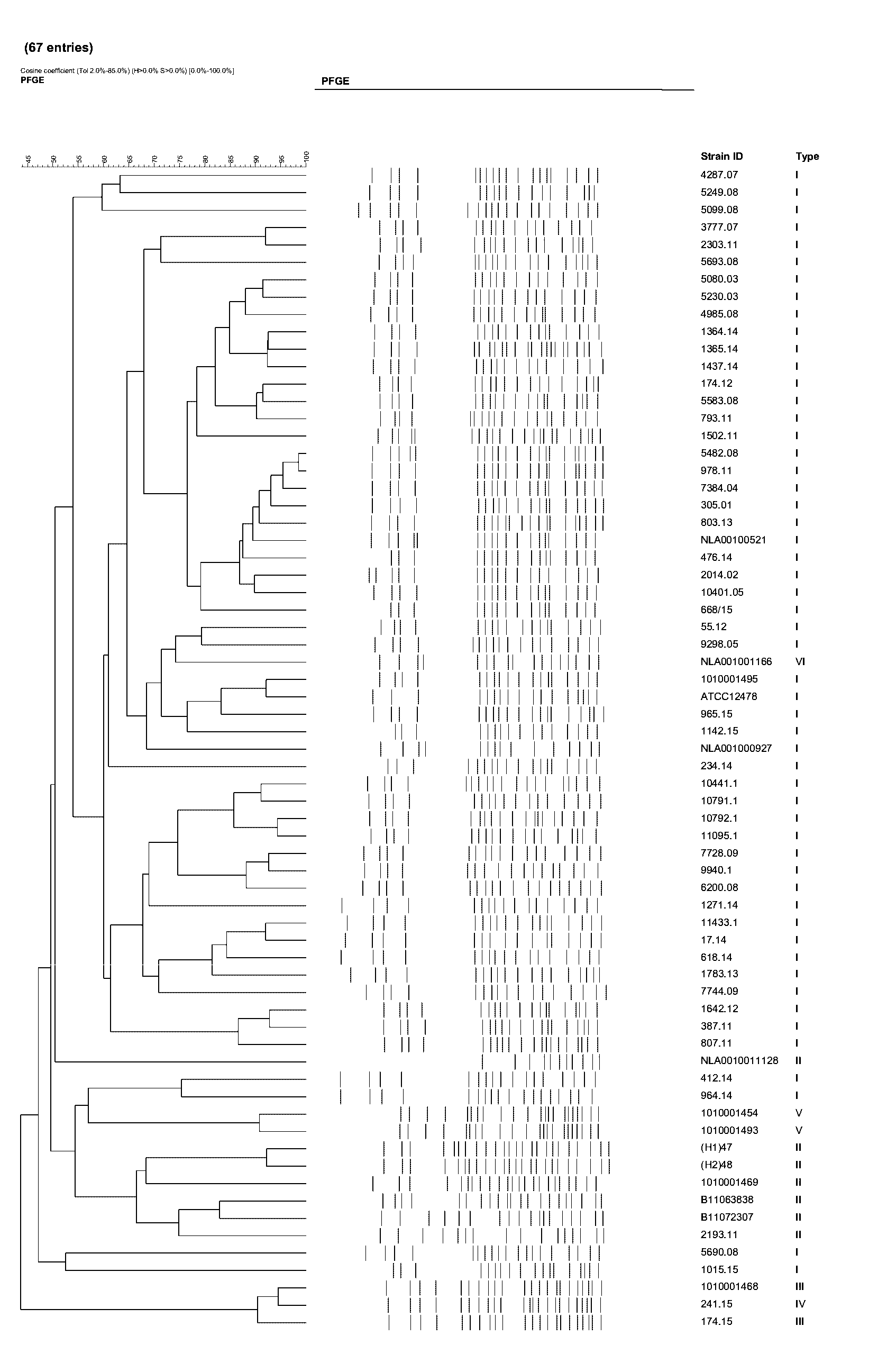


**SUPPLEMENTARY FIGURE 2. VNTR typing.** A dendrogram for cluster analysis was based on the obtained VNTR profiles and constructed according to Pearson correlation algorithm. NP, no PCR product; MP, multiple-band profile. **
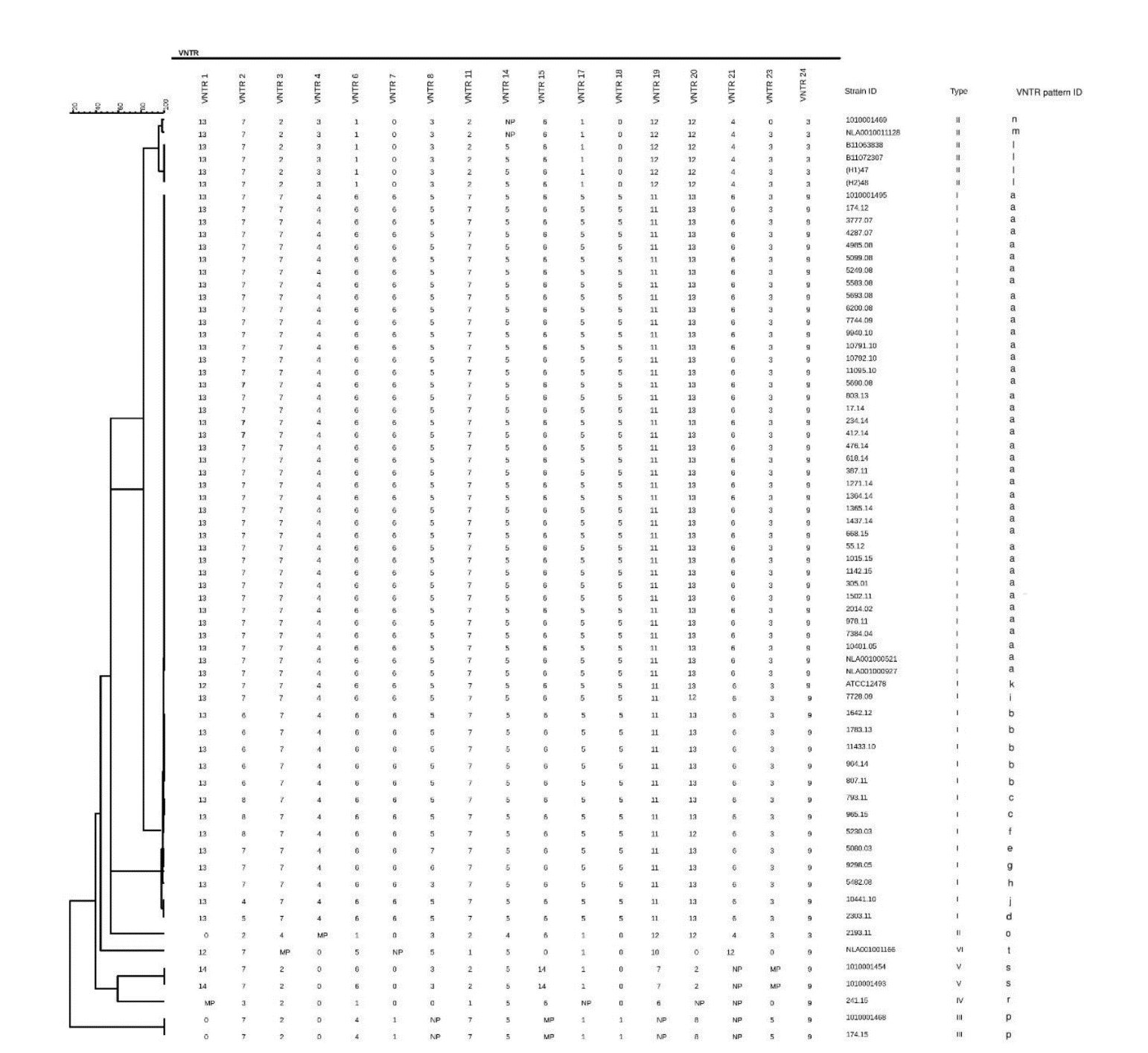
**

**SUPPLEMENTARY TABLES**

**SUPPLEMENTARY TABLE 1.** *Mycobacterium kansasii* isolates used in the study.

| **No.** | **Isolate ID** | **Type** | **NTM disease** | **Country** | **Source** | **CODE** | |
| --- | --- | --- | --- | --- | --- | --- | --- |
|  |  |  |  |  |  | **PFGE** | **VNTR** |
|  | 305.01 | I | no | Poland | Sputum | H | a |
|  | 9940.01 |  | yes | Poland | Sputum | S | a |
|  | 10441.01 |  | yes | Poland | Aspiration material | R | j |
|  | 10791.01 |  | no | Poland | Sputum | R | a |
|  | 10792.01 |  | no | Poland | Sputum | R | a |
|  | 11095.01 |  | no | Poland | Sputum | R | a |
|  | 11433.01 |  | no | Poland | Sputum | U | b |
|  | 2014.02 |  | no | Poland | Sputum | H | a |
|  | 5080.03 |  | no | Poland | Sputum | F | e |
|  | 5230.03 |  | yes | Poland | Sputum | F | f |
|  | 7384.04 |  | yes | Poland | Bronchial washings | H | a |
|  | 9298.05 |  | yes | Poland | Sputum | K | g |
|  | 10401.05 |  | no | Poland | Sputum | H | a |
|  | 3777.07 |  | no | Poland | Sputum | D | a |
|  | 4287.07 |  | no | Poland | Sputum | A | a |
|  | 4985.08 |  | no | Poland | Sputum | F | a |
|  | 5099.08 |  | no | Poland | Sputum | C | a |
|  | 5249.08 |  | no | Poland | Sputum | B | a |
|  | 5482.08 |  | yes | Poland | Bronchial washings | H | h |
|  | 5583.08 |  | yes | Poland | Bronchial washings | F | a |
|  | 5693.08 |  | yes | Poland | Sputum | E | a |
|  | 6200.08 |  | yes | Poland | Sputum | S | a |
|  | 5690.08 |  | no | Poland | Bronchial washings | AG | a |
|  | 7728.09 |  | yes | Poland | Bronchial washings | S | i |
|  | 7744.09 |  | yes | Poland | Bronchial washings | W | a |
|  | 1502.11 |  | yes | Poland | Bronchoalveolar lavage fluid | H | a |
|  | 978.11 |  | yes | Poland | Sputum | G | a |
|  | 807.11 |  | yes | Poland | Sputum | H | a |
|  | 387.11 |  | yes | Poland | Sputum | X | b |
|  | 793.11 |  | yes | Poland | Bronchial washings | X | a |
|  | 2303.11 |  | no | Poland | Sputum | F | c |
|  | 55.12 |  | yes | Poland | Bronchial washings | D | d |
|  | 1642.12 |  | no | Poland | Sputum | J | a |
|  | 174.12 |  | yes | Poland | Bronchial washings | X | b |
|  | 803.13 |  | yes | Poland | Bronchial washings | F | a |
|  | 1783.13 |  | no | Poland | Sputum | H | a |
|  | 17.14 |  | yes | Poland | Sputum | U | b |
|  | 234.14 |  | yes | Poland | Bronchial washings | U | a |
|  | 412.14 |  | yes | Poland | Sputum | P | a |
|  | 476.14 |  | yes | Poland | Bronchial washings | Z | a |
|  | 618.14 |  | yes | Poland | Bronchial washings | H | a |
|  | 964.14 |  | no | Poland | Sputum | U | a |
|  | 1271.14 |  | yes | Poland | Sputum | AA | b |
|  | 1364.14 |  | yes | Poland | Sputum | T | a |
|  | 1365.14 |  | yes | Poland | Sputum | F | a |
|  | 1437.14 |  | yes | Poland | Bronchial washings | F | a |
|  | 668.15 |  | yes | Poland | Bronchoalveolar lavage fluid | F | a |
|  | 965.15 |  | yes | Poland | Bronchial washings | I | a |
|  | 1015.15 |  | yes | Poland | Sputum | M | c |
|  | 1142.15 |  | yes | Poland | Bronchoalveolar lavage fluid | AH | a |
|  | NLA00100521 |  | yes | the Netherlands | Sputum | H | a |
|  | NLA001000927 |  | yes | the Netherlands | Sputum | O | a |
|  | 1010001495 |  | -^a^ | the Czech Republic | Water | M | a |
|  | ATCC12478 |  | yes | Reference Strain | Clinical | M | k |
|  | 2193.11 | II | no | Poland | Bronchial washings | AF | o |
|  | B11072307 |  | no | the Netherlands | Sputum | AE | l |
|  | B11063838 |  | no | the Netherlands | Bronchoalveolar lavage fluid | AE | l |
|  | NLA0010011128 |  | no | the Netherlands | Bronchoalveolar lavage fluid | Y | m |
|  | 1010001469 |  | - | Italy | Water | AD | n |
|  | (H1)47 |  | yes | Spain | Sputum | AC | l |
|  | (H2)48 |  | yes | Spain | Sputum | AC | l |
|  | 1010001468 | III | - | Belgium | Soil | AI | p |
|  | 174.15 |  | no | the Czech Republic | Sputum | AI | p |
|  | 241.15 | IV | no | the Czech Republic | Sputum | AI | r |
|  | 1010001454 | V | - | Germany | Water | AB | s |
|  | 1010001493 |  | - | the Netherlands | Water | AB | s |
|  | NLA001001166 | VI | no | the Netherlands | Sputum | L | t |

^a^ Environmental strain.

**SUPPLEMENTARY TABLE 2.** Characteristics of selected VNTR loci identified in the genome of the *M. kansasii* ATCC 12478 reference strain.

| **VNTR locus** | | **Repeat length x copy no. (+flanking sequence)^a^** | **Location of VNTR^b^** | | **Repeat consensus sequence** | **ORF in ATCC 12478^c^** | **Designed primer sequence (5’- 3’)^d^** | **Annealing temperature** |
| --- | --- | --- | --- | --- | --- | --- | --- | --- |
|  |  |  | **Start** | **End** |  |  |  |  |
| **LONG REPEATS** | **1** | 56 bp x 11.9 (+121 bp) | 5532142 | 5532809 | TGATTGGCGTCGACCCGCCGCGCCCCGCTTCGCGGGGCTTGCGATCGCCGCTGGAT | [thymidylate synthase](https://www.ncbi.nlm.nih.gov/nucleotide/556021639?report=gbwithparts&from=5532821&to=5533624&RID=7ESEXSCY013) | F: ATGGCCTCCGTTGTACTCCTGCGG  R: TCGGATTTGGCCGAACCCGTT | 66ºC |
|  | **2** | 68 bp x 6.7 (+130 bp) | 509237 | 509689 | GCGGCCCGCGTAGCGGGCCGTTGAGGAGGCGGGCAATCGGGCTAGGCCCGCCGACCCACAACTTC | Pup deamidase/depupylase | F: CTCGACCTCCGTGCCGATAATC  R: CAACTGAATGCGCCAGTCGATC | 60ºC |
|  | **3** | 63 bp x 6.7 (+395 bp) | 1275514 | 1275926 | ATTTGATTCGTGTTGGCAGCGACCCGCAGCGCCCGACTCCGTCGCGCTTGCGATCGCTGCCG | short-chain dehydrogenase | F: CCAACCCAGGCCATAGGTAACCG  R: GGGTATCGGCATGCGCCAATC | 63ºC |
|  | **4** | 77 bp x 4.5 (+425 bp) | 4018462 | 4018810 | GATGACCCGCGCCGACGACGATGCAGTGCGAAGCAATGAGGTGGGGGTACCGCCCGCTTGCGGGGGAGAGTGGCGCT | histidine kinase | F: ACAACGGGAGCATCGGCGTGTGGA  R: TCTCGCTGTCCCGAGCCGTCACCAT | 66ºC |
|  | **5** | 57 bp x 5.9 (+304 bp) | 4752931 | 4753264 | GTGGCGATCGCAAGCTCGGCGGAGCCGGGCGTGGCGGGTCGCCGCGTGGTGGGTTCC | [2-isopropylmalate synthase](https://www.ncbi.nlm.nih.gov/nucleotide/556021639?report=gbwithparts&from=4752931&to=4753278&RID=7EU348H5013) | F: GATTGCCGAACGCGAGGTGGAG  R: CCGCAGTAGCGCCGCATCGGCCCA | 63ºC |
|  | **6** | 56 bp x 6.3 (+407 bp) | 5717225 | 5717547 | TGAGTTGCGGCGGCGACGATGCAGAGCGCAGCGATGAGGAGGAGCGGCGCGCA | diadenosine polyphosphate  hydrolase | F: AGGCGATTCGAGTCATCAAGACGG  R: CCCGTCCAACATGTCGAAAAGCAC | 65ºC |
|  | **7** | 55 bp x 5.8 (+503 bp) | 2629164 | 2629480 | TCGCGACCCGCTGCGCCCGGCCCTGCCGCGCTTGCGATCGCTCCTAACCCTATGT | acetaldehyde dehydrogenase | F: CCCCTCCTTTTTCGACCAACGCA  R: GACATCGTATTGCCCGGATCCTGC | 65ºC |
|  | **8** | 57 bp x 5.1 (+311 bp) | 3872114 | 3872403 | AGCCCGACGACGGTGAGCGCCGAGACGGCGCGAGTAAGGAGTCGGGCAATCAGACCG | thioesterase | F: TGCACGTCGACTACCGCAAGATCAC  R: GGCGAATACATCCGAGAGGCCACC | 64ºC |
|  | **9** | 54 bp x 5 (+554 bp) | 2007710 | 2007977 | GTGGCGATCGCGAGCGCGGCGCAGCCGGGCGAAGCGGGTCGCCACCATCGGCCG | enoyl-CoA hydratase | F: ACCCACACCGGCGGCACCTTC  R: GCTTCCTCCGAAACGAACAGCCG | 66ºC |
|  | **10** | 54 bp x 8.5 (+200 bp) | 5186966 | 5187424 | GCCCGGCTGCGCCGGGCTCGCGACCGTCTGGGTCCGATGACGACGGCCCGCAGC | MFS transporter | F: CGCAATCGCGACCATCGACCAG  R: CGATTCGACCAAGGCGCATTCG | 66ºC |
|  | **11** | 55 bp x 6.9 (+492 bp) | 1243727 | 1244104 | AACTTGCCCCACTCCTCAACGGCCGCACCGGCCGCATCGTCGTGGGACTGTGCCT | NAD-glutamate  dehydrogenase/ABC transporter ATP-binding protein | F: ACAGCTACACAGCTCTCACCGGCC  R: CTTCCCTGGCTGTGCTGTGGTGA | 64ºC |
|  | **12** | 57 bp x 5.3 (+122 bp) | 1434953 | 1435252 | GCGCTTGCGATCGCCGCGGTGCTTGACGAGCGGCGACCCGCCGCGCCCGGCTTGGCC | homoserine kinase | F: CAGGCAGTGACTGGGTCACCAACAG  R: CGGCGCTCAAGGACATGCCG | 63ºC |
|  | **13** | 56 bp x 5 (+361 bp) | 3284931 | 3285209 | TGATGGGCCGCGGCTCTCTTCGCGCGCTGGCGCGCGCTCATCGCCCGCTGCCGGGT | NF | F: GCCGACAAGTGGGAAGCGCTTG  R: GATTTCTGTTTCCTGGTCACCCAGCG | 64ºC |
|  | **14** | 87 bp x 4.6 (+213 bp) | 212288 | 212683 | CGGGAACAGCGTGATGCCATTCACCGGATCACCGACAACACCACCCACATTGATACCCAACGGAATCCCAGGAAGCGTATAACCACC | NF | F: ACATGAAGGCTCAACGGGATCTGG  R: CGATTACGATTCCCCAGTCTCCCTT | 60ºC |
|  | **15** | 56 bp x 5.6 (+200 bp) | 4728932 | 4729256 | GCCGTGCGACGATCCGGGACGGTGGGGCCCGTTGAGGAGCGCGGCAATTGGGCTGC | DeoR family transcriptional  regulator | F: GAGTTGTAGCCGGCTCTTGAGCG  R: CGAGTGTGGATGTAGGGCGAGACT | 65ºC |
|  | **16** | 54 bp x 4.6 (+413 bp) | 2409378 | 2409628 | CGATCGCAAGCGCGGCAGTGCCGCGCGCAGCGGGTCGCCACCAGGCGTCACCGC | NF | F: CCGGTTCGGGCAGATCCATCAG  R: CGGTGGGAGCGGTCGACGAATAC | 64ºC |
|  | **17** | 52 bp x 5 (+437 bp) | 137195 | 137503 | GCCGGATCGGTCGTCTCGCTCCCGTCACCCACTGCGTTCGTTCCGCTCGCTCGACTCCCAGG | TetR family transcriptional  regulator/phosphoketolase | F: AGCTCTTATGCGACTGACACGGAG  R: ATCTTCGCCTACCACGGGTATCC | 65ºC |
| **SHORT REPEATS** | **18** | 40 bp x 5.1 (+186 bp) | 5591855 | 5592137 | TCGGCCCGGCGACGATGCAGGCTTTGCCTGTTGAGGAGCCGGGCGAATGTGGT | polyphosphate glucokinase/  inositol monophosphatase | F: GGCGAGAGACGTATGTGTCCGA  R: GTCGGATCGTGTCACCGCTCTA | 60ºC |
|  | **19** | 18 bp x 10.7 (+154 bp) | 6055108 | 6055300 | GCCGTTGCCGTTGGTGTG | membrane protein | F: ATGTCGACGGCGGACAACG  R: GACCGCATCCAACACTCCCTG | 62ºC |
|  | **20** | 20 bp x 12.8 (+256 bp) | 5796131 | 5796386 | CGCCACCAGTGCACGCTCGC | NF | F: GGGCATCGAGCAGGCAGC  R: GACCGAGTTGCGCAGCAGG | 65ºC |
|  | **21** | 24 bp x 5.6 (+187 bp) | 4095156 | 4095289 | CCGCCGACGACGCCACCCACCACG | membrane protein | F: GCCTCCCGGTGTCGTACCC  R: GGAACGGTCGTCGGAGCG | 65ºC |
|  | **22** | 21 bp x 16.8 (+459 bp) | 2155137 | 2155489 | GCCGACCTGGCACCCGCACCT | resuscitation-promoting factor RpfA | F: GCCTCCCACGGCGGCGGCGAGTTC  R: ACCTGGTGGATTTCCACCGGTGC | 64ºC |
|  | **23** | 40 bp x 5.1 (+430 bp) | 2263403 | 2263607 | GTGCTTCGACGGCGTCCAGTGTGCGCTGACGGCGCCGAGT | short-chain dehydrogenase | F: ACAGCGACCGGGCTATTTTGACG  R: AACATGTCGAGCTACCTCACCGGC | 65ºC |
|  | **24** | 20 bp x 8.7 (+192 bp) | 5896379 | 5896552 | GCACACTCGGCGCCAGGAGC | esterase | F: CGATGACGGCTTTGAGTGTGCGT  R: GTAGTTGCGGGGGTCCGACATTTAG | 62ºC |

Grey color indicates VNTR loci excluded from the analysis due to the lack of PCR product or multiple-band profiles;

^a^ Full-length consensus repeat in *M. kansasii* ATCC 12478. Number of repeats was calculated as a quotient of the VNTR loci length to the size of the repeat consensus;

^b^ Position in *M. kansasii* ATCC 12478 genome;

^c^ Annotation based on the NCBI Prokaryotic Genome Annotation Pipeline (2013). NF, not found or hypothetical protein;

^d^ F, forward primer; R, reverse primer.

**SUPPLEMENTARY TABLE 3.** Allele calling table.

| **No. of repeats** | **VNTR locus** | | | | | | | | | | | | | | | | |
| --- | --- | --- | --- | --- | --- | --- | --- | --- | --- | --- | --- | --- | --- | --- | --- | --- | --- |
|  | **1** | **2** | **3** | **4** | **6** | **7** | **8** | **11** | **14** | **15** | **17** | **18** | **19** | **20** | **21** | **23** | **24** |
|  | 56^a^ | 68 | 63 | 77 | 56 | 55 | 57 | 55 | 87 | 56 | 52 | 40 | 18 | 20 | 24 | 40 | 20 |
|  | 221^b^ | 130 | 395 | 425 | 407 | 503 | 311 | 492 | 213 | 200 | 437 | 186 | 186 | 256 | 187 | 330 | 192 |
|  | 11.9^c^ | 6.7 | 6.7 | 4.5 | 6.3 | 5.8 | 5.1 | 6.9 | 4.6 | 5.8 | 5 | 5.1 | 10.7 | 12.8 | 5.6 | 5.1 | 8.7 |
| **0** | <271 | <177 | <177 | <540 | <480 | <547 | <374 | <541 | <265 | <244 | <489 | <230 | <166 | <281 | <201 | <374 | <206 |
| **1** | 271 | 177 | 439 | 540 | 480 | 547 | 374 | 541 | 265 | 244 | 489 | 230 | 166 | 281 | 201 | 374 | 206 |
| **2** | 327 | 245 | 502 | 617 | 536 | 602 | 431 | 596 | 352 | 300 | 541 | 270 | 184 | 301 | 225 | 414 | 226 |
| **3** | 383 | 313 | 565 | 694 | 592 | 657 | 488 | 651 | 439 | 356 | 593 | 310 | 202 | 321 | 249 | 454 | 246 |
| **4** | 439 | 381 | 628 | **771** | 648 | 712 | 545 | 706 | 526 | 412 | 645 | 350 | 220 | 341 | 273 | 494 | 266 |
| **5** | 495 | 449 | 691 | 848 | 704 | 767 | **602** | 761 | **613** | 468 | **697** | **390** | 238 | 361 | 297 | **534** | 286 |
| **6** | 551 | 517 | 754 | 925 | **760** | **822** | 659 | 816 | 700 | **524** | 749 | 430 | 256 | 381 | **321** | 574 | 306 |
| **7** | 607 | **585** | **817** | 1002 | 816 | 877 | 716 | **871** | 787 | 580 | 801 | 470 | 274 | 401 | 345 | 614 | 326 |
| **8** | 663 | 653 | 880 | 1079 | 872 | 932 | 773 | 926 | 874 | 636 | 853 | 510 | 292 | 421 | 369 | 654 | 346 |
| **9** | 719 | 721 | 943 | 1156 | 928 | 987 | 830 | 981 | 961 | 692 | 905 | 550 | 310 | 441 | 393 | 694 | **366** |
| **10** | 775 | 789 | 1006 | 1233 | 984 | 1042 | 887 | 1036 | 1048 | 748 | 957 | 590 | 328 | 461 | 417 | 734 | 386 |
| **11** | 831 | 857 | 1069 | 1310 | 1040 | 1097 | 944 | 1091 | 1135 | 804 | 1009 | 630 | **346** | 481 | 441 | 774 | 406 |
| **12** | **887** | 925 | 1132 | 1387 | 1096 | 1152 | 1001 | 1146 | 1222 | 860 | 1061 | 670 | 364 | 501 | 465 | 814 | 426 |
| **13** | 943 | 993 | 1195 | 1464 | 1152 | 1207 | 1058 | 1201 | 1309 | 916 | 1113 | 710 | 382 | **521** | 489 | 854 | 446 |
| **14** | 999 | 1061 | 1258 | 1541 | 1208 | 1262 | 1115 | 1256 | 1396 | 972 | 1165 | 750 | 400 | 541 | 513 | 894 | 466 |
| **15** | 1055 | 1129 | 1321 | 1618 | 1264 | 1317 | 1172 | 1311 | 1483 | 1028 | 1217 | 790 | 418 | 561 | 537 | 934 | 486 |
| **16** | 1111 | 1197 | 1384 | 1695 | 1320 | 1372 | 1229 | 1366 | 1570 | 1084 | 1269 | 830 | 436 | 581 | 561 | 974 | 506 |
| **17** | 1167 | 1265 | 1447 | 1772 | 1376 | 1427 | 1286 | 1421 | 1657 | 1140 | 1321 | 870 | 454 | 601 | 585 | 1014 | 526 |
| **18** | 1223 | 1333 | 1510 | 1849 | 1432 | 1482 | 1343 | 1476 | 1744 | 1196 | 1373 | 910 | 472 | 621 | 609 | 1054 | 546 |
| **19** | 1279 | 1401 | 1573 | 1926 | 1488 | 1537 | 1400 | 1531 | 1831 | 1252 | 1425 | 950 | 490 | 641 | 633 | 1094 | 566 |
| **20** | 1335 | 1469 | 1636 | 2003 | 1544 | 1592 | 1457 | 1586 | 1918 | 1308 | 1477 | 990 | 508 | 661 | 657 | 1134 | 586 |

Grey color indicates product length [bp] for *M. kansasii* ATCC 12478;

^a^ Full-length consensus repeat [bp] in *M. kansasii* ATCC 12478;

^b^ Flanking sequence length [bp] in *M. kansasii* ATCC 12478;

^c^ Total number of repeats in *M. kansasii* ATCC 12478.

**SUPPLEMENTARY TABLE 4.** Characteristics of all VNTR loci identified in the genome of the *M. kansasii* ATCC 12478 reference strain.

| **Location of VNTR^a^** | | **Fragment length** | **Repeat length** | **Copy number** | **% of conservation**  **between TRs** |
| --- | --- | --- | --- | --- | --- |
| **Start** | **End** |  |  |  |  |
| 5532142 | 5532809 | 667 | 56 | 11.9 | 100 |
| 509237 | 509689 | 452 | 68 | 6.7 | 100 |
| 1275514 | 1275926 | 412 | 62 | 6.7 | 100 |
| 1576443 | 1576500 | 57 | 9 | 6.4 | 100 |
| 5717225 | 5717547 | 322 | 53 | 6.1 | 100 |
| 6153787 | 6153858 | 71 | 12 | 6.0 | 100 |
| 4752931 | 4753264 | 333 | 57 | 5.9 | 100 |
| 2629164 | 2629480 | 316 | 55 | 5.8 | 100 |
| 2718092 | 2718160 | 68 | 12 | 5.8 | 100 |
| 4737548 | 4737581 | 33 | 6 | 5.7 | 100 |
| 1545745 | 1545851 | 106 | 20 | 5.3 | 100 |
| 4671907 | 4671980 | 73 | 14 | 5.3 | 100 |
| 3872114 | 3872403 | 289 | 57 | 5.1 | 100 |
| 3511155 | 3511205 | 50 | 10 | 5.1 | 100 |
| 2007710 | 2007977 | 267 | 54 | 5.0 | 100 |
| 2691824 | 2691883 | 59 | 12 | 5.0 | 100 |
| 2996643 | 2996691 | 48 | 10 | 4.9 | 100 |
| 6149658 | 6149705 | 47 | 10 | 4.8 | 100 |
| 2607541 | 2607569 | 28 | 6 | 4.8 | 100 |
| 4737515 | 4737543 | 28 | 6 | 4.8 | 100 |
| 3460765 | 3460815 | 50 | 11 | 4.6 | 100 |
| 4018462 | 4018810 | 348 | 77 | 4.5 | 100 |
| 3033690 | 3033779 | 89 | 20 | 4.5 | 100 |
| 947315 | 947359 | 44 | 10 | 4.5 | 100 |
| 85545 | 85595 | 50 | 12 | 4.3 | 100 |
| 6217570 | 6217791 | 221 | 53 | 4.2 | 100 |
| 4049254 | 4049480 | 226 | 56 | 4.1 | 100 |
| 770641 | 770685 | 44 | 11 | 4.1 | 100 |
| 261573 | 261609 | 36 | 9 | 4.1 | 100 |
| 2040944 | 2040980 | 36 | 9 | 4.1 | 100 |
| 4079136 | 4079178 | 42 | 11 | 3.9 | 100 |
| 943151 | 943185 | 34 | 9 | 3.9 | 100 |
| 85714 | 85758 | 44 | 12 | 3.8 | 100 |
| 5585111 | 5585152 | 41 | 11 | 3.8 | 100 |
| 74644 | 74677 | 33 | 9 | 3.8 | 100 |
| 169084 | 169117 | 33 | 9 | 3.8 | 100 |
| 1080803 | 1080839 | 36 | 10 | 3.7 | 100 |
| 2598883 | 2598915 | 32 | 9 | 3.7 | 100 |
| 2601361 | 2601393 | 32 | 9 | 3.7 | 100 |
| 2601715 | 2601747 | 32 | 9 | 3.7 | 100 |
| 2602777 | 2602809 | 32 | 9 | 3.7 | 100 |
| 6226056 | 6226088 | 32 | 9 | 3.7 | 100 |
| 6095342 | 6095545 | 203 | 56 | 3.6 | 100 |
| 1613811 | 1613850 | 39 | 11 | 3.6 | 100 |
| 1166127 | 1166162 | 35 | 10 | 3.6 | 100 |
| 3641275 | 3641479 | 204 | 58 | 3.5 | 100 |
| 5413272 | 5413476 | 204 | 58 | 3.5 | 100 |
| 2750988 | 2751175 | 187 | 55 | 3.4 | 100 |
| 3606682 | 3606732 | 50 | 15 | 3.4 | 100 |
| 2455272 | 2455305 | 33 | 10 | 3.4 | 100 |
| 2355146 | 2355176 | 30 | 9 | 3.4 | 100 |
| 5058925 | 5058955 | 30 | 9 | 3.4 | 100 |
| 5423481 | 5423511 | 30 | 9 | 3.4 | 100 |
| 2331188 | 2331220 | 32 | 10 | 3.3 | 100 |
| 3519181 | 3519213 | 32 | 10 | 3.3 | 100 |
| 942187 | 942216 | 29 | 9 | 3.3 | 100 |
| 2645494 | 2645523 | 29 | 9 | 3.3 | 100 |
| 2709780 | 2709809 | 29 | 9 | 3.3 | 100 |
| 2804631 | 2804660 | 29 | 9 | 3.3 | 100 |
| 3847249 | 3847283 | 34 | 11 | 3.2 | 100 |
| 105465 | 105496 | 31 | 10 | 3.2 | 100 |
| 4265770 | 4265801 | 31 | 10 | 3.2 | 100 |
| 48978 | 49006 | 28 | 9 | 3.2 | 100 |
| 2341536 | 2341564 | 28 | 9 | 3.2 | 100 |
| 1823191 | 1823369 | 178 | 57 | 3.1 | 100 |
| 3553294 | 3553459 | 165 | 54 | 3.1 | 100 |
| 296615 | 296642 | 27 | 9 | 3.1 | 100 |
| 1763083 | 1763110 | 27 | 9 | 3.1 | 100 |
| 5676017 | 5676044 | 27 | 9 | 3.1 | 100 |
| 5735559 | 5735586 | 27 | 9 | 3.1 | 100 |
| 5735985 | 5736012 | 27 | 9 | 3.1 | 100 |
| 3598559 | 3598627 | 68 | 23 | 3.0 | 100 |
| 4095459 | 4095503 | 44 | 15 | 3.0 | 100 |
| 393030 | 393059 | 29 | 10 | 3.0 | 100 |
| 3041858 | 3041887 | 29 | 10 | 3.0 | 100 |
| 8348 | 8374 | 26 | 9 | 3.0 | 100 |
| 729050 | 729076 | 26 | 9 | 3.0 | 100 |
| 1591600 | 1591626 | 26 | 9 | 3.0 | 100 |
| 1673302 | 1673328 | 26 | 9 | 3.0 | 100 |
| 1794488 | 1794514 | 26 | 9 | 3.0 | 100 |
| 5458473 | 5458499 | 26 | 9 | 3.0 | 100 |
| 5924069 | 5924095 | 26 | 9 | 3.0 | 100 |
| 4365459 | 4365498 | 39 | 14 | 2.9 | 100 |
| 4671050 | 4671081 | 31 | 11 | 2.9 | 100 |
| 2878061 | 2878089 | 28 | 10 | 2.9 | 100 |
| 1763533 | 1763558 | 25 | 9 | 2.9 | 100 |
| 2645708 | 2645733 | 25 | 9 | 2.9 | 100 |
| 3918224 | 3918249 | 25 | 9 | 2.9 | 100 |
| 5638301 | 5638326 | 25 | 9 | 2.9 | 100 |
| 1568639 | 1568798 | 159 | 57 | 2.8 | 100 |
| 5465711 | 5465869 | 158 | 56 | 2.8 | 100 |
| 4005589 | 4005713 | 124 | 44 | 2.8 | 100 |
| 5847515 | 5847551 | 36 | 13 | 2.8 | 100 |
| 6263573 | 6263609 | 36 | 13 | 2.8 | 100 |
| 4125972 | 4126005 | 33 | 12 | 2.8 | 100 |
| 14000 | 14024 | 24 | 9 | 2.8 | 100 |
| 74836 | 74860 | 24 | 9 | 2.8 | 100 |
| 1543061 | 1543085 | 24 | 9 | 2.8 | 100 |
| 1735789 | 1735813 | 24 | 9 | 2.8 | 100 |
| 1763406 | 1763430 | 24 | 9 | 2.8 | 100 |
| 1765148 | 1765172 | 24 | 9 | 2.8 | 100 |
| 1825381 | 1825405 | 24 | 9 | 2.8 | 100 |
| 2644389 | 2644413 | 24 | 9 | 2.8 | 100 |
| 2810009 | 2810033 | 24 | 9 | 2.8 | 100 |
| 3180425 | 3180449 | 24 | 9 | 2.8 | 100 |
| 2021356 | 2021541 | 185 | 69 | 2.7 | 100 |
| 4094651 | 4094699 | 48 | 18 | 2.7 | 100 |
| 5047297 | 5047328 | 31 | 12 | 2.7 | 100 |
| 5576689 | 5576718 | 29 | 11 | 2.7 | 100 |
| 1825421 | 1825574 | 153 | 59 | 2.6 | 100 |
| 2122626 | 2122654 | 28 | 11 | 2.6 | 100 |
| 386065 | 386090 | 25 | 10 | 2.6 | 100 |
| 4547970 | 4548106 | 136 | 54 | 2.5 | 100 |
| 2304979 | 2305013 | 34 | 14 | 2.5 | 100 |
| 4384118 | 4384147 | 29 | 12 | 2.5 | 100 |
| 2179810 | 2179837 | 27 | 11 | 2.5 | 100 |
| 4237762 | 4237786 | 24 | 10 | 2.5 | 100 |
| 2355110 | 2355152 | 42 | 18 | 2.4 | 100 |
| 203650 | 203690 | 40 | 18 | 2.3 | 100 |
| 5539816 | 5539856 | 40 | 18 | 2.3 | 100 |
| 341031 | 341058 | 27 | 12 | 2.3 | 100 |
| 2573713 | 2573740 | 27 | 12 | 2.3 | 100 |
| 3787817 | 3787844 | 27 | 12 | 2.3 | 100 |
| 4491588 | 4491615 | 27 | 12 | 2.3 | 100 |
| 2659800 | 2659826 | 26 | 12 | 2.3 | 100 |
| 4649974 | 4650000 | 26 | 12 | 2.3 | 100 |
| 4935583 | 4935609 | 26 | 12 | 2.3 | 100 |
| 5577006 | 5577032 | 26 | 12 | 2.3 | 100 |
| 5957512 | 5957641 | 129 | 58 | 2.2 | 100 |
| 4326600 | 4326625 | 25 | 12 | 2.2 | 100 |
| 5388113 | 5388138 | 25 | 12 | 2.2 | 100 |
| 5559722 | 5559783 | 61 | 30 | 2.1 | 100 |
| 2881811 | 2881862 | 51 | 25 | 2.1 | 100 |
| 3344013 | 3344047 | 34 | 17 | 2.1 | 100 |
| 1861311 | 1861344 | 33 | 16 | 2.1 | 100 |
| 3138476 | 3138508 | 32 | 16 | 2.1 | 100 |
| 1171148 | 1171178 | 30 | 15 | 2.1 | 100 |
| 215348 | 215376 | 28 | 14 | 2.1 | 100 |
| 1550917 | 1550943 | 26 | 13 | 2.1 | 100 |
| 1262824 | 1262848 | 24 | 12 | 2.1 | 100 |
| 2064962 | 2064986 | 24 | 12 | 2.1 | 100 |
| 2684904 | 2684928 | 24 | 12 | 2.1 | 100 |
| 2982309 | 2982333 | 24 | 12 | 2.1 | 100 |
| 4408408 | 4408432 | 24 | 12 | 2.1 | 100 |
| 4481985 | 4482009 | 24 | 12 | 2.1 | 100 |
| 4678998 | 4679022 | 24 | 12 | 2.1 | 100 |
| 5362438 | 5362462 | 24 | 12 | 2.1 | 100 |
| 5605783 | 5605829 | 46 | 24 | 2.0 | 100 |
| 6154390 | 6154436 | 46 | 24 | 2.0 | 100 |
| 6316696 | 6316739 | 43 | 22 | 2.0 | 100 |
| 4648316 | 4648358 | 42 | 22 | 2.0 | 100 |
| 27401 | 27441 | 40 | 20 | 2.0 | 100 |
| 4637930 | 4637970 | 40 | 20 | 2.0 | 100 |
| 201094 | 201133 | 39 | 20 | 2.0 | 100 |
| 3328843 | 3328881 | 38 | 20 | 2.0 | 100 |
| 5410633 | 5410668 | 35 | 18 | 2.0 | 100 |
| 940240 | 940269 | 29 | 15 | 2.0 | 100 |
| 5712844 | 5712869 | 25 | 13 | 2.0 | 100 |
| 2948464 | 2948533 | 69 | 36 | 1.9 | 100 |
| 3315072 | 3315128 | 56 | 30 | 1.9 | 100 |
| 1839231 | 1839268 | 37 | 20 | 1.9 | 100 |
| 3574053 | 3574087 | 34 | 18 | 1.9 | 100 |
| 3892509 | 3892543 | 34 | 18 | 1.9 | 100 |
| 4369018 | 4369052 | 34 | 18 | 1.9 | 100 |
| 870157 | 870185 | 28 | 15 | 1.9 | 100 |
| 2064846 | 2064874 | 28 | 15 | 1.9 | 100 |
| 3487389 | 3487417 | 28 | 15 | 1.9 | 100 |
| 3775034 | 3775062 | 28 | 15 | 1.9 | 100 |
| 4956922 | 4956950 | 28 | 15 | 1.9 | 100 |
| 5043083 | 5043111 | 28 | 15 | 1.9 | 100 |
| 5407954 | 5407982 | 28 | 15 | 1.9 | 100 |
| 6154417 | 6154445 | 28 | 15 | 1.9 | 100 |
| 1576829 | 1576853 | 24 | 13 | 1.9 | 100 |
| 5683716 | 5683740 | 24 | 13 | 1.9 | 100 |
| 5186966 | 5187424 | 458 | 54 | 8.5 | 99 |
| 1243727 | 1244104 | 377 | 55 | 6.9 | 99 |
| 1434953 | 1435252 | 299 | 57 | 5.3 | 99 |
| 3284931 | 3285209 | 278 | 56 | 5.0 | 99 |
| 3309951 | 3310131 | 180 | 57 | 3.2 | 99 |
| 323515 | 324779 | 1264 | 432 | 2.9 | 99 |
| 5363037 | 5363225 | 188 | 66 | 2.9 | 99 |
| 432895 | 433948 | 1053 | 527 | 2.0 | 99 |
| 448891 | 449944 | 1053 | 527 | 2.0 | 99 |
| 1778152 | 1778269 | 117 | 10 | 11.7 | 98 |
| 2567412 | 2567471 | 59 | 9 | 6.7 | 98 |
| 3924384 | 3924447 | 63 | 10 | 6.4 | 98 |
| 4728932 | 4729256 | 324 | 56 | 5.8 | 98 |
| 1490309 | 1490372 | 63 | 13 | 4.9 | 98 |
| 212288 | 212683 | 395 | 87 | 4.6 | 98 |
| 2409378 | 2409628 | 250 | 54 | 4.6 | 98 |
| 308995 | 309275 | 280 | 66 | 4.3 | 98 |
| 5218727 | 5218933 | 206 | 54 | 3.8 | 98 |
| 190267 | 190335 | 68 | 18 | 3.8 | 98 |
| 6213601 | 6214381 | 780 | 243 | 3.2 | 98 |
| 2385615 | 2385933 | 318 | 106 | 3.0 | 98 |
| 4548112 | 4548203 | 91 | 36 | 2.6 | 98 |
| 1935782 | 1936080 | 298 | 122 | 2.5 | 98 |
| 2750776 | 2750908 | 132 | 55 | 2.4 | 98 |
| 2903163 | 2903257 | 94 | 39 | 2.4 | 98 |
| 3394581 | 3394714 | 133 | 61 | 2.2 | 98 |
| 210739 | 211030 | 291 | 138 | 2.1 | 98 |
| 2385615 | 2385933 | 318 | 54 | 6.0 | 97 |
| 2884836 | 2884885 | 49 | 9 | 5.6 | 97 |
| 5334072 | 5334126 | 54 | 10 | 5.5 | 97 |
| 4633671 | 4633719 | 48 | 9 | 5.4 | 97 |
| 4766694 | 4766742 | 48 | 9 | 5.4 | 97 |
| 137195 | 137503 | 308 | 62 | 5.0 | 97 |
| 2248462 | 2248520 | 58 | 12 | 5.0 | 97 |
| 210204 | 210508 | 304 | 69 | 4.4 | 97 |
| 4393723 | 4393873 | 150 | 44 | 3.4 | 97 |
| 2939601 | 2939714 | 113 | 36 | 3.2 | 97 |
| 190570 | 190626 | 56 | 18 | 3.2 | 97 |
| 21079 | 21595 | 516 | 165 | 3.1 | 97 |
| 1019429 | 1019603 | 174 | 57 | 3.1 | 97 |
| 852399 | 852470 | 71 | 24 | 3.0 | 97 |
| 3915676 | 3915816 | 140 | 48 | 2.9 | 97 |
| 2189782 | 2189916 | 134 | 60 | 2.3 | 97 |
| 4061019 | 4061080 | 61 | 27 | 2.3 | 97 |
| 6002992 | 6003158 | 166 | 75 | 2.2 | 97 |
| 6120077 | 6120160 | 83 | 39 | 2.2 | 97 |
| 2663843 | 2663909 | 66 | 30 | 2.2 | 97 |
| 2189816 | 2190010 | 194 | 93 | 2.1 | 97 |
| 3913323 | 3913406 | 83 | 40 | 2.1 | 97 |
| 2598509 | 2606462 | 7953 | 354 | 22.5 | 96 |
| 6055108 | 6055300 | 192 | 18 | 10.7 | 96 |
| 4364968 | 4365034 | 66 | 10 | 6.7 | 96 |
| 1561047 | 1561118 | 71 | 12 | 6.0 | 96 |
| 5591855 | 5592137 | 282 | 53 | 5.3 | 96 |
| 4668823 | 4668947 | 124 | 27 | 4.6 | 96 |
| 790665 | 790742 | 77 | 18 | 4.3 | 96 |
| 2639233 | 2639462 | 229 | 57 | 4.0 | 96 |
| 4280870 | 4280904 | 34 | 9 | 4.0 | 96 |
| 3288660 | 3288787 | 127 | 33 | 3.9 | 96 |
| 2228880 | 2228918 | 38 | 10 | 3.9 | 96 |
| 5735844 | 5735878 | 34 | 9 | 3.9 | 96 |
| 1944050 | 1944093 | 43 | 12 | 3.8 | 96 |
| 1672511 | 1672544 | 33 | 9 | 3.8 | 96 |
| 2055481 | 2055524 | 43 | 12 | 3.7 | 96 |
| 3913398 | 3913472 | 74 | 21 | 3.6 | 96 |
| 5315923 | 5315958 | 35 | 10 | 3.6 | 96 |
| 4878350 | 4878386 | 36 | 11 | 3.4 | 96 |
| 74154 | 74340 | 186 | 56 | 3.3 | 96 |
| 695115 | 695153 | 38 | 12 | 3.3 | 96 |
| 2952980 | 2953018 | 38 | 12 | 3.3 | 96 |
| 1746102 | 1746147 | 45 | 15 | 3.1 | 96 |
| 2750856 | 2751044 | 188 | 66 | 2.9 | 96 |
| 3758387 | 3758427 | 40 | 14 | 2.9 | 96 |
| 3014907 | 3014944 | 37 | 13 | 2.9 | 96 |
| 17553 | 17987 | 434 | 165 | 2.6 | 96 |
| 4543480 | 4543525 | 45 | 18 | 2.6 | 96 |
| 3178432 | 3178488 | 56 | 24 | 2.4 | 96 |
| 544826 | 544869 | 43 | 18 | 2.4 | 96 |
| 3951871 | 3951926 | 55 | 24 | 2.3 | 96 |
| 2385615 | 2385933 | 318 | 160 | 2.0 | 96 |
| 545294 | 545346 | 52 | 27 | 2.0 | 96 |
| 1033783 | 1033835 | 52 | 27 | 2.0 | 96 |
| 2597688 | 2597744 | 56 | 30 | 1.9 | 96 |
| 784642 | 784721 | 79 | 12 | 6.7 | 95 |
| 1759178 | 1759227 | 49 | 9 | 5.6 | 95 |
| 2386254 | 2386353 | 99 | 20 | 5.0 | 95 |
| 3371480 | 3371603 | 123 | 26 | 4.9 | 95 |
| 1205572 | 1205629 | 57 | 12 | 4.8 | 95 |
| 2570303 | 2570356 | 53 | 12 | 4.5 | 95 |
| 645668 | 645865 | 197 | 51 | 3.8 | 95 |
| 2598529 | 2598561 | 32 | 9 | 3.7 | 95 |
| 2599237 | 2599269 | 32 | 9 | 3.7 | 95 |
| 2599591 | 2599623 | 32 | 9 | 3.7 | 95 |
| 2599945 | 2599977 | 32 | 9 | 3.7 | 95 |
| 2600299 | 2600331 | 32 | 9 | 3.7 | 95 |
| 2600653 | 2600685 | 32 | 9 | 3.7 | 95 |
| 2601007 | 2601039 | 32 | 9 | 3.7 | 95 |
| 2602069 | 2602101 | 32 | 9 | 3.7 | 95 |
| 2602423 | 2602455 | 32 | 9 | 3.7 | 95 |
| 2603131 | 2603163 | 32 | 9 | 3.7 | 95 |
| 2603485 | 2603517 | 32 | 9 | 3.7 | 95 |
| 2603839 | 2603871 | 32 | 9 | 3.7 | 95 |
| 2604193 | 2604225 | 32 | 9 | 3.7 | 95 |
| 2604547 | 2604579 | 32 | 9 | 3.7 | 95 |
| 2604902 | 2604934 | 32 | 9 | 3.7 | 95 |
| 2605254 | 2605286 | 32 | 9 | 3.7 | 95 |
| 2605608 | 2605640 | 32 | 9 | 3.7 | 95 |
| 2605962 | 2605994 | 32 | 9 | 3.7 | 95 |
| 1672844 | 1672875 | 31 | 9 | 3.6 | 95 |
| 4617541 | 4617572 | 31 | 9 | 3.6 | 95 |
| 6004174 | 6004205 | 31 | 9 | 3.6 | 95 |
| 5817571 | 5817601 | 30 | 9 | 3.6 | 95 |
| 2127786 | 2127922 | 136 | 42 | 3.3 | 95 |
| 14304 | 14333 | 29 | 9 | 3.3 | 95 |
| 296464 | 296493 | 29 | 9 | 3.3 | 95 |
| 1768628 | 1768657 | 29 | 9 | 3.3 | 95 |
| 2735106 | 2735135 | 29 | 9 | 3.3 | 95 |
| 2795640 | 2795669 | 29 | 9 | 3.3 | 95 |
| 4309199 | 4309228 | 29 | 9 | 3.3 | 95 |
| 5662965 | 5662999 | 34 | 11 | 3.2 | 95 |
| 886799 | 886829 | 30 | 10 | 3.1 | 95 |
| 3415933 | 3415963 | 30 | 10 | 3.1 | 95 |
| 1396647 | 1396709 | 62 | 21 | 3.0 | 95 |
| 2048668 | 2048697 | 29 | 10 | 3.0 | 95 |
| 5737469 | 5737500 | 31 | 11 | 2.9 | 95 |
| 57236 | 57267 | 31 | 12 | 2.8 | 95 |
| 1226683 | 1226713 | 30 | 11 | 2.8 | 95 |
| 3137744 | 3137774 | 30 | 11 | 2.8 | 95 |
| 4887104 | 4887555 | 451 | 165 | 2.7 | 95 |
| 2300481 | 2300512 | 31 | 12 | 2.7 | 95 |
| 5075972 | 5076045 | 73 | 30 | 2.5 | 95 |
| 5077247 | 5077320 | 73 | 30 | 2.5 | 95 |
| 5078531 | 5078604 | 73 | 30 | 2.5 | 95 |
| 3838392 | 3838463 | 71 | 30 | 2.4 | 95 |
| 518740 | 518863 | 123 | 55 | 2.3 | 95 |
| 6000841 | 6000881 | 40 | 18 | 2.3 | 95 |
| 5079539 | 5079573 | 34 | 15 | 2.3 | 95 |
| 4369018 | 4369056 | 38 | 18 | 2.2 | 95 |
| 1165896 | 1165976 | 80 | 39 | 2.1 | 95 |
| 4465777 | 4465814 | 37 | 18 | 2.1 | 95 |
| 5933481 | 5933518 | 37 | 18 | 2.1 | 95 |
| 5839430 | 5841443 | 2013 | 993 | 2.0 | 95 |
| 5046939 | 5047030 | 91 | 45 | 2.0 | 95 |
| 5744337 | 5744383 | 46 | 24 | 2.0 | 95 |
| 2725468 | 2725510 | 42 | 21 | 2.0 | 95 |
| 2138302 | 2138342 | 40 | 20 | 2.0 | 95 |
| 3394883 | 3394923 | 40 | 20 | 2.0 | 95 |
| 6357460 | 6357499 | 39 | 20 | 2.0 | 95 |
| 3394550 | 3394696 | 146 | 79 | 1.9 | 95 |
| 5796131 | 5796386 | 255 | 20 | 12.8 | 94 |
| 4095156 | 4095289 | 133 | 24 | 5.6 | 94 |
| 2866769 | 2866816 | 47 | 9 | 5.3 | 94 |
| 6345164 | 6345210 | 46 | 9 | 5.2 | 94 |
| 2124205 | 2124369 | 164 | 40 | 4.1 | 94 |
| 3426567 | 3426611 | 44 | 11 | 4.1 | 94 |
| 6153886 | 6154018 | 132 | 39 | 3.4 | 94 |
| 5917849 | 5917968 | 119 | 36 | 3.3 | 94 |
| 537327 | 537384 | 57 | 20 | 2.9 | 94 |
| 3898876 | 3898905 | 29 | 11 | 2.7 | 94 |
| 987944 | 987974 | 30 | 12 | 2.6 | 94 |
| 5918768 | 5918798 | 30 | 12 | 2.6 | 94 |
| 1878119 | 1878148 | 29 | 12 | 2.5 | 94 |
| 3371480 | 3371603 | 123 | 51 | 2.4 | 94 |
| 6320696 | 6320995 | 299 | 129 | 2.3 | 94 |
| 562339 | 562372 | 33 | 15 | 2.3 | 94 |
| 1946841 | 1946873 | 32 | 15 | 2.2 | 94 |
| 2295954 | 2295986 | 32 | 15 | 2.2 | 94 |
| 3775034 | 3775066 | 32 | 15 | 2.2 | 94 |
| 485677 | 485713 | 36 | 18 | 2.1 | 94 |
| 1840176 | 1840212 | 36 | 18 | 2.1 | 94 |
| 6304776 | 6304812 | 36 | 18 | 2.1 | 94 |
| 1551309 | 1551343 | 34 | 17 | 2.1 | 94 |
| 756947 | 756978 | 31 | 15 | 2.1 | 94 |
| 2281384 | 2281415 | 31 | 15 | 2.1 | 94 |
| 2575797 | 2575827 | 30 | 15 | 2.1 | 94 |
| 548162 | 548197 | 35 | 18 | 2.0 | 94 |
| 5933281 | 5933316 | 35 | 18 | 2.0 | 94 |
| 6325517 | 6325552 | 35 | 18 | 2.0 | 94 |
| 1561229 | 1561262 | 33 | 17 | 2.0 | 94 |
| 4890697 | 4891268 | 571 | 297 | 1.9 | 94 |
| 5585144 | 5585183 | 39 | 21 | 1.9 | 94 |
| 173717 | 173754 | 37 | 20 | 1.9 | 94 |
| 8100 | 8134 | 34 | 18 | 1.9 | 94 |
| 1239233 | 1239267 | 34 | 18 | 1.9 | 94 |
| 5839468 | 5839502 | 34 | 18 | 1.9 | 94 |
| 5840461 | 5840495 | 34 | 18 | 1.9 | 94 |
| 2155137 | 2155489 | 352 | 21 | 16.8 | 93 |
| 1592121 | 1592245 | 124 | 9 | 13.9 | 93 |
| 5896379 | 5896552 | 173 | 20 | 8.7 | 93 |
| 5822015 | 5822123 | 108 | 20 | 5.5 | 93 |
| 2263403 | 2263607 | 204 | 40 | 5.1 | 93 |
| 3503904 | 3503944 | 40 | 9 | 4.6 | 93 |
| 5355851 | 5355891 | 40 | 9 | 4.6 | 93 |
| 551887 | 551926 | 39 | 9 | 4.4 | 93 |
| 4617603 | 4617642 | 39 | 9 | 4.4 | 93 |
| 3913323 | 3913407 | 84 | 20 | 4.3 | 93 |
| 3640929 | 3640971 | 42 | 10 | 4.3 | 93 |
| 1674061 | 1674099 | 38 | 9 | 4.3 | 93 |
| 4617599 | 4617636 | 37 | 9 | 4.3 | 93 |
| 210725 | 211003 | 278 | 69 | 4.0 | 93 |
| 475798 | 475876 | 78 | 20 | 4.0 | 93 |
| 4992481 | 4992525 | 44 | 12 | 3.8 | 93 |
| 203525 | 203588 | 63 | 18 | 3.6 | 93 |
| 1517327 | 1517460 | 133 | 40 | 3.4 | 93 |
| 2910347 | 2910436 | 89 | 30 | 3.0 | 93 |
| 4548550 | 4548680 | 130 | 45 | 2.9 | 93 |
| 3394806 | 3394858 | 52 | 20 | 2.6 | 93 |
| 3400826 | 3400882 | 56 | 24 | 2.4 | 93 |
| 4307622 | 4307681 | 59 | 27 | 2.2 | 93 |
| 3773657 | 3773776 | 119 | 57 | 2.1 | 93 |
| 1668222 | 1668339 | 117 | 57 | 2.1 | 93 |
| 554621 | 554651 | 30 | 15 | 2.1 | 93 |
| 3270461 | 3270491 | 30 | 15 | 2.1 | 93 |
| 3417529 | 3417559 | 30 | 15 | 2.1 | 93 |
| 4125996 | 4126026 | 30 | 15 | 2.1 | 93 |
| 3721800 | 3721829 | 29 | 15 | 2.1 | 93 |
| 4429657 | 4429715 | 58 | 30 | 2.0 | 93 |
| 1924126 | 1924155 | 29 | 15 | 2.0 | 93 |
| 2051071 | 2051100 | 29 | 15 | 2.0 | 93 |
| 3806529 | 3806558 | 29 | 15 | 2.0 | 93 |
| 4329607 | 4329636 | 29 | 15 | 2.0 | 93 |
| 5058317 | 5058346 | 29 | 15 | 2.0 | 93 |
| 5805263 | 5805292 | 29 | 15 | 2.0 | 93 |
| 360929 | 360957 | 28 | 14 | 2.0 | 93 |
| 2356470 | 2356498 | 28 | 15 | 2.0 | 93 |
| 3801074 | 3801341 | 267 | 144 | 1.9 | 93 |
| 5746237 | 5746266 | 29 | 16 | 1.9 | 93 |
| 1517336 | 1517452 | 116 | 20 | 5.8 | 92 |
| 2925653 | 2925714 | 61 | 12 | 5.2 | 92 |
| 4016288 | 4016324 | 36 | 9 | 4.0 | 92 |
| 2598037 | 2598071 | 34 | 9 | 3.9 | 92 |
| 2733559 | 2733593 | 34 | 9 | 3.9 | 92 |
| 2735322 | 2735356 | 34 | 9 | 3.9 | 92 |
| 2646072 | 2646105 | 33 | 9 | 3.8 | 92 |
| 3573092 | 3573125 | 33 | 9 | 3.8 | 92 |
| 3705685 | 3705718 | 33 | 9 | 3.8 | 92 |
| 3757682 | 3757715 | 33 | 9 | 3.8 | 92 |
| 3542285 | 3542318 | 33 | 9 | 3.7 | 92 |
| 1223669 | 1223744 | 75 | 21 | 3.6 | 92 |
| 2948283 | 2948336 | 53 | 15 | 3.6 | 92 |
| 5719174 | 5719211 | 37 | 11 | 3.5 | 92 |
| 20008 | 20570 | 562 | 165 | 3.4 | 92 |
| 3605762 | 3605870 | 108 | 32 | 3.4 | 92 |
| 3572905 | 3572964 | 59 | 18 | 3.3 | 92 |
| 2589404 | 2589442 | 38 | 12 | 3.3 | 92 |
| 2589408 | 2589446 | 38 | 12 | 3.3 | 92 |
| 877350 | 877411 | 61 | 20 | 3.1 | 92 |
| 5896290 | 5896347 | 57 | 20 | 2.9 | 92 |
| 4890863 | 4892279 | 1416 | 510 | 2.8 | 92 |
| 3839735 | 3839774 | 39 | 15 | 2.7 | 92 |
| 1935862 | 1936276 | 414 | 160 | 2.6 | 92 |
| 5355846 | 5355891 | 45 | 18 | 2.6 | 92 |
| 3157108 | 3157326 | 218 | 90 | 2.5 | 92 |
| 190358 | 190425 | 67 | 27 | 2.5 | 92 |
| 475290 | 475879 | 589 | 254 | 2.3 | 92 |
| 5470264 | 5470389 | 125 | 56 | 2.3 | 92 |
| 5936341 | 5936461 | 120 | 53 | 2.3 | 92 |
| 5079839 | 5079908 | 69 | 30 | 2.3 | 92 |
| 2051455 | 2051628 | 173 | 87 | 2.0 | 92 |
| 546041 | 546094 | 53 | 27 | 2.0 | 92 |
| 645668 | 645865 | 197 | 108 | 1.9 | 92 |
| 5081492 | 5081548 | 56 | 30 | 1.9 | 92 |
| 475028 | 475440 | 412 | 20 | 20.3 | 91 |
| 4125928 | 4125978 | 50 | 6 | 8.5 | 91 |
| 1014246 | 1014300 | 54 | 9 | 6.1 | 91 |
| 212923 | 213294 | 371 | 72 | 5.2 | 91 |
| 4568396 | 4568475 | 79 | 20 | 4.0 | 91 |
| 1165895 | 1165973 | 78 | 20 | 4.0 | 91 |
| 2239078 | 2239188 | 110 | 30 | 3.7 | 91 |
| 295894 | 295926 | 32 | 9 | 3.7 | 91 |
| 1672786 | 1672818 | 32 | 9 | 3.7 | 91 |
| 561131 | 561239 | 108 | 30 | 3.6 | 91 |
| 896985 | 897016 | 31 | 9 | 3.6 | 91 |
| 2354266 | 2354297 | 31 | 9 | 3.6 | 91 |
| 784642 | 784721 | 79 | 24 | 3.3 | 91 |
| 5785402 | 5785433 | 31 | 10 | 3.1 | 91 |
| 2757137 | 2757208 | 71 | 24 | 3.0 | 91 |
| 5243798 | 5243831 | 33 | 11 | 3.0 | 91 |
| 555529 | 555614 | 85 | 30 | 2.9 | 91 |
| 2607792 | 2607844 | 52 | 18 | 2.9 | 91 |
| 624958 | 624992 | 34 | 12 | 2.9 | 91 |
| 1718480 | 1718514 | 34 | 12 | 2.9 | 91 |
| 3802349 | 3802473 | 124 | 45 | 2.8 | 91 |
| 2412755 | 2412788 | 33 | 12 | 2.8 | 91 |
| 3832744 | 3832782 | 38 | 15 | 2.6 | 91 |
| 3836965 | 3837003 | 38 | 15 | 2.6 | 91 |
| 5476112 | 5476149 | 37 | 15 | 2.5 | 91 |
| 980182 | 980243 | 61 | 27 | 2.3 | 91 |
| 3572905 | 3572966 | 61 | 27 | 2.3 | 91 |
| 1533158 | 1533199 | 41 | 18 | 2.3 | 91 |
| 2606711 | 2606752 | 41 | 18 | 2.3 | 91 |
| 1576401 | 1576441 | 40 | 18 | 2.3 | 91 |
| 5427874 | 5427938 | 64 | 30 | 2.2 | 91 |
| 547025 | 547065 | 40 | 18 | 2.2 | 91 |
| 4553859 | 4553901 | 42 | 20 | 2.1 | 91 |
| 2770080 | 2770121 | 41 | 19 | 2.1 | 91 |
| 695133 | 695203 | 70 | 36 | 2.0 | 91 |
| 3399122 | 3399168 | 46 | 24 | 2.0 | 91 |
| 3605752 | 3605870 | 118 | 11 | 11.0 | 90 |
| 3750201 | 3750421 | 220 | 20 | 10.9 | 90 |
| 475544 | 475696 | 152 | 20 | 7.6 | 90 |
| 4061018 | 4061080 | 62 | 9 | 6.9 | 90 |
| 3571568 | 3571628 | 60 | 9 | 6.8 | 90 |
| 2910349 | 2910436 | 87 | 15 | 5.9 | 90 |
| 2767468 | 2767572 | 104 | 18 | 5.8 | 90 |
| 2732728 | 2732777 | 49 | 9 | 5.6 | 90 |
| 2770080 | 2770121 | 41 | 10 | 4.2 | 90 |
| 3288639 | 3288775 | 136 | 33 | 4.1 | 90 |
| 3371113 | 3371431 | 318 | 80 | 4.0 | 90 |
| 1947529 | 1947613 | 84 | 21 | 4.0 | 90 |
| 131132 | 131175 | 43 | 11 | 4.0 | 90 |
| 2413190 | 2413260 | 70 | 18 | 3.9 | 90 |
| 3542780 | 3542820 | 40 | 11 | 3.8 | 90 |
| 2341558 | 2341624 | 66 | 18 | 3.7 | 90 |
| 2547964 | 2548005 | 41 | 12 | 3.5 | 90 |
| 672015 | 672056 | 41 | 12 | 3.4 | 90 |
| 1122752 | 1122782 | 30 | 9 | 3.4 | 90 |
| 2646264 | 2646293 | 29 | 9 | 3.3 | 90 |
| 4892480 | 4892706 | 226 | 72 | 3.2 | 90 |
| 3640942 | 3641072 | 130 | 42 | 3.2 | 90 |
| 4859746 | 4859838 | 92 | 30 | 3.1 | 90 |
| 3775082 | 3775143 | 61 | 21 | 3.0 | 90 |
| 4990981 | 4991013 | 32 | 11 | 3.0 | 90 |
| 2598778 | 2598827 | 49 | 18 | 2.8 | 90 |
| 2602318 | 2602367 | 49 | 18 | 2.8 | 90 |
| 2604797 | 2604846 | 49 | 18 | 2.8 | 90 |
| 2605503 | 2605552 | 49 | 18 | 2.8 | 90 |
| 2605857 | 2605906 | 49 | 18 | 2.8 | 90 |
| 2239715 | 2239795 | 80 | 30 | 2.7 | 90 |
| 3641176 | 3641280 | 104 | 40 | 2.6 | 90 |
| 3394684 | 3394749 | 65 | 25 | 2.6 | 90 |
| 3801233 | 3801504 | 271 | 108 | 2.5 | 90 |
| 790665 | 790754 | 89 | 36 | 2.5 | 90 |
| 1946846 | 1946882 | 36 | 15 | 2.5 | 90 |
| 1149218 | 1149250 | 32 | 13 | 2.4 | 90 |
| 3110530 | 3110634 | 104 | 45 | 2.3 | 90 |
| 3821509 | 3821544 | 35 | 15 | 2.3 | 90 |
| 2412754 | 2412788 | 34 | 15 | 2.3 | 90 |
| 3128499 | 3128533 | 34 | 15 | 2.3 | 90 |
| 21455 | 21722 | 267 | 120 | 2.2 | 90 |
| 3130392 | 3130450 | 58 | 27 | 2.2 | 90 |
| 8189 | 8228 | 39 | 18 | 2.2 | 90 |
| 1459465 | 1459504 | 39 | 18 | 2.2 | 90 |
| 6153550 | 6153589 | 39 | 18 | 2.2 | 90 |
| 545807 | 545845 | 38 | 18 | 2.2 | 90 |
| 549109 | 549147 | 38 | 18 | 2.2 | 90 |
| 2780311 | 2780349 | 38 | 18 | 2.2 | 90 |
| 5781987 | 5782025 | 38 | 18 | 2.2 | 90 |
| 5090006 | 5090042 | 36 | 18 | 2.2 | 90 |
| 22591 | 22653 | 62 | 30 | 2.1 | 90 |
| 2732974 | 2733011 | 37 | 18 | 2.1 | 90 |
| 1143479 | 1143515 | 36 | 17 | 2.1 | 90 |
| 2780854 | 2780890 | 36 | 18 | 2.1 | 90 |
| 2633841 | 2633927 | 86 | 45 | 2.0 | 90 |
| 6120001 | 6120061 | 60 | 30 | 2.0 | 90 |
| 3174232 | 3174272 | 40 | 21 | 2.0 | 90 |
| 3775004 | 3775044 | 40 | 21 | 2.0 | 90 |
| 2646453 | 2646498 | 45 | 24 | 1.9 | 90 |
| 886865 | 886909 | 44 | 23 | 1.9 | 90 |
| 1872358 | 1872399 | 41 | 22 | 1.9 | 90 |
| 3801695 | 3801999 | 304 | 36 | 8.5 | 89 |
| 4094654 | 4094699 | 45 | 9 | 5.1 | 89 |
| 1894972 | 1895052 | 80 | 18 | 4.5 | 89 |
| 1759178 | 1759254 | 76 | 18 | 4.3 | 89 |
| 6304778 | 6304815 | 37 | 9 | 4.2 | 89 |
| 5422667 | 5422703 | 36 | 9 | 4.1 | 89 |
| 215583 | 215767 | 184 | 51 | 3.6 | 89 |
| 130225 | 130332 | 107 | 30 | 3.6 | 89 |
| 3487644 | 3487682 | 38 | 12 | 3.3 | 89 |
| 1021978 | 1022092 | 114 | 40 | 2.9 | 89 |
| 5075363 | 5078949 | 3586 | 1284 | 2.8 | 89 |
| 2189782 | 2189949 | 167 | 60 | 2.8 | 89 |
| 4095601 | 4095685 | 84 | 30 | 2.8 | 89 |
| 5722686 | 5722770 | 84 | 30 | 2.8 | 89 |
| 715959 | 715988 | 29 | 11 | 2.7 | 89 |
| 2598688 | 2598734 | 46 | 18 | 2.6 | 89 |
| 2599042 | 2599088 | 46 | 18 | 2.6 | 89 |
| 2599396 | 2599442 | 46 | 18 | 2.6 | 89 |
| 2599750 | 2599796 | 46 | 18 | 2.6 | 89 |
| 2600104 | 2600150 | 46 | 18 | 2.6 | 89 |
| 2600458 | 2600504 | 46 | 18 | 2.6 | 89 |
| 2600812 | 2600858 | 46 | 18 | 2.6 | 89 |
| 2601166 | 2601212 | 46 | 18 | 2.6 | 89 |
| 2601520 | 2601566 | 46 | 18 | 2.6 | 89 |
| 2601874 | 2601920 | 46 | 18 | 2.6 | 89 |
| 2602228 | 2602274 | 46 | 18 | 2.6 | 89 |
| 2602582 | 2602628 | 46 | 18 | 2.6 | 89 |
| 2602936 | 2602982 | 46 | 18 | 2.6 | 89 |
| 2603290 | 2603336 | 46 | 18 | 2.6 | 89 |
| 2603644 | 2603690 | 46 | 18 | 2.6 | 89 |
| 2603998 | 2604044 | 46 | 18 | 2.6 | 89 |
| 2604352 | 2604398 | 46 | 18 | 2.6 | 89 |
| 2604707 | 2604753 | 46 | 18 | 2.6 | 89 |
| 2605061 | 2605107 | 46 | 18 | 2.6 | 89 |
| 2605413 | 2605459 | 46 | 18 | 2.6 | 89 |
| 2605767 | 2605813 | 46 | 18 | 2.6 | 89 |
| 2606121 | 2606167 | 46 | 18 | 2.6 | 89 |
| 3371092 | 3371140 | 48 | 20 | 2.5 | 89 |
| 3732321 | 3732369 | 48 | 20 | 2.5 | 89 |
| 1654333 | 1654380 | 47 | 20 | 2.4 | 89 |
| 2124185 | 2124369 | 184 | 80 | 2.3 | 89 |
| 2433774 | 2433841 | 67 | 30 | 2.3 | 89 |
| 323740 | 323773 | 33 | 15 | 2.3 | 89 |
| 324172 | 324205 | 33 | 15 | 2.3 | 89 |
| 324604 | 324637 | 33 | 15 | 2.3 | 89 |
| 2412973 | 2413006 | 33 | 15 | 2.3 | 89 |
| 4853924 | 4853957 | 33 | 15 | 2.3 | 89 |
| 1517327 | 1517460 | 133 | 60 | 2.2 | 89 |
| 85552 | 85604 | 52 | 24 | 2.2 | 89 |
| 547037 | 547073 | 36 | 18 | 2.1 | 89 |
| 1520762 | 1520798 | 36 | 18 | 2.1 | 89 |
| 2181087 | 2181123 | 36 | 18 | 2.1 | 89 |
| 5735559 | 5735595 | 36 | 18 | 2.1 | 89 |
| 323659 | 323717 | 58 | 30 | 2.0 | 89 |
| 323842 | 323900 | 58 | 30 | 2.0 | 89 |
| 324091 | 324149 | 58 | 30 | 2.0 | 89 |
| 324274 | 324332 | 58 | 30 | 2.0 | 89 |
| 324523 | 324581 | 58 | 30 | 2.0 | 89 |
| 6095201 | 6095239 | 38 | 20 | 2.0 | 89 |
| 4466438 | 4466473 | 35 | 18 | 2.0 | 89 |
| 3300517 | 3300574 | 57 | 30 | 1.9 | 89 |
| 549699 | 549738 | 39 | 21 | 1.9 | 89 |
| 1695888 | 1695924 | 36 | 19 | 1.9 | 89 |
| 1894964 | 1895048 | 84 | 9 | 9.4 | 88 |
| 4019968 | 4020035 | 67 | 9 | 7.6 | 88 |
| 2127786 | 2127922 | 136 | 21 | 6.5 | 88 |
| 1021973 | 1022092 | 119 | 20 | 6.0 | 88 |
| 5734999 | 5735051 | 52 | 9 | 5.9 | 88 |
| 5782146 | 5782196 | 50 | 9 | 5.7 | 88 |
| 203641 | 203683 | 42 | 9 | 4.8 | 88 |
| 1457010 | 1457052 | 42 | 9 | 4.8 | 88 |
| 4020163 | 4020205 | 42 | 9 | 4.8 | 88 |
| 5194146 | 5194267 | 121 | 27 | 4.6 | 88 |
| 115380 | 115424 | 44 | 11 | 4.1 | 88 |
| 5279991 | 5280026 | 35 | 9 | 4.0 | 88 |
| 886865 | 886909 | 44 | 12 | 3.9 | 88 |
| 3704797 | 3704831 | 34 | 9 | 3.9 | 88 |
| 5932881 | 5932915 | 34 | 9 | 3.9 | 88 |
| 5781891 | 5781924 | 33 | 9 | 3.8 | 88 |
| 5918092 | 5918186 | 94 | 36 | 2.6 | 88 |
| 4375102 | 4375155 | 53 | 21 | 2.6 | 88 |
| 4737541 | 4737585 | 44 | 18 | 2.5 | 88 |
| 5179089 | 5179135 | 46 | 20 | 2.4 | 88 |
| 1457258 | 1457301 | 43 | 18 | 2.4 | 88 |
| 2040948 | 2040991 | 43 | 18 | 2.4 | 88 |
| 5918208 | 5918250 | 42 | 18 | 2.4 | 88 |
| 4766694 | 4766755 | 61 | 27 | 2.3 | 88 |
| 203630 | 203690 | 60 | 27 | 2.3 | 88 |
| 4729262 | 4729306 | 44 | 20 | 2.3 | 88 |
| 784642 | 784721 | 79 | 36 | 2.2 | 88 |
| 558160 | 558225 | 65 | 30 | 2.2 | 88 |
| 2355201 | 2355246 | 45 | 21 | 2.2 | 88 |
| 1129311 | 1129379 | 68 | 33 | 2.1 | 88 |
| 3834243 | 3834306 | 63 | 30 | 2.1 | 88 |
| 77695 | 77727 | 32 | 15 | 2.1 | 88 |
| 4484186 | 4484218 | 32 | 15 | 2.1 | 88 |
| 627691 | 627739 | 48 | 24 | 2.0 | 88 |
| 549519 | 549554 | 35 | 18 | 2.0 | 88 |
| 549720 | 549755 | 35 | 18 | 2.0 | 88 |
| 666434 | 666469 | 35 | 18 | 2.0 | 88 |
| 1943093 | 1943128 | 35 | 18 | 2.0 | 88 |
| 3573822 | 3573857 | 35 | 18 | 2.0 | 88 |
| 3835841 | 3835897 | 56 | 30 | 1.9 | 88 |
| 4553960 | 4553997 | 37 | 20 | 1.9 | 88 |
| 2341536 | 2341570 | 34 | 18 | 1.9 | 88 |
| 2597986 | 2598020 | 34 | 18 | 1.9 | 88 |
| 3072896 | 3072930 | 34 | 18 | 1.9 | 88 |
| 5457902 | 5457936 | 34 | 18 | 1.9 | 88 |
| 5715810 | 5715844 | 34 | 18 | 1.9 | 88 |
| 5735662 | 5735696 | 34 | 18 | 1.9 | 88 |
| 5899862 | 5899895 | 33 | 18 | 1.9 | 88 |
| 778664 | 778696 | 32 | 16 | 1.9 | 88 |
| 1630370 | 1630402 | 32 | 17 | 1.9 | 88 |
| 2529020 | 2529052 | 32 | 17 | 1.9 | 88 |
| 4548550 | 4548680 | 130 | 15 | 8.7 | 87 |
| 2153803 | 2153877 | 74 | 12 | 6.3 | 87 |
| 2580294 | 2580342 | 48 | 10 | 4.9 | 87 |
| 2079781 | 2079822 | 41 | 9 | 4.7 | 87 |
| 548078 | 548118 | 40 | 9 | 4.6 | 87 |
| 2925608 | 2925647 | 39 | 9 | 4.6 | 87 |
| 4977071 | 4977110 | 39 | 9 | 4.4 | 87 |
| 5745004 | 5745106 | 102 | 24 | 4.3 | 87 |
| 1763304 | 1763342 | 38 | 9 | 4.3 | 87 |
| 2607110 | 2607148 | 38 | 9 | 4.3 | 87 |
| 4243200 | 4243243 | 43 | 12 | 3.7 | 87 |
| 16496 | 17054 | 558 | 165 | 3.4 | 87 |
| 1203188 | 1203234 | 46 | 15 | 3.1 | 87 |
| 5917695 | 5917854 | 159 | 54 | 3.0 | 87 |
| 1070497 | 1070582 | 85 | 30 | 2.9 | 87 |
| 1433175 | 1433235 | 60 | 21 | 2.9 | 87 |
| 2254960 | 2255010 | 50 | 18 | 2.8 | 87 |
| 2604088 | 2604138 | 50 | 18 | 2.8 | 87 |
| 2606211 | 2606261 | 50 | 18 | 2.8 | 87 |
| 4527100 | 4527149 | 49 | 18 | 2.8 | 87 |
| 3833082 | 3833159 | 77 | 30 | 2.6 | 87 |
| 3837303 | 3837380 | 77 | 30 | 2.6 | 87 |
| 3573147 | 3573195 | 48 | 18 | 2.6 | 87 |
| 2098262 | 2098300 | 38 | 15 | 2.6 | 87 |
| 2101055 | 2101093 | 38 | 15 | 2.6 | 87 |
| 2103875 | 2103913 | 38 | 15 | 2.6 | 87 |
| 3571561 | 3571628 | 67 | 27 | 2.5 | 87 |
| 1668279 | 1668384 | 105 | 45 | 2.4 | 87 |
| 5081165 | 5081235 | 70 | 30 | 2.4 | 87 |
| 6320152 | 6320451 | 299 | 129 | 2.3 | 87 |
| 4885268 | 4885336 | 68 | 30 | 2.3 | 87 |
| 1141271 | 1141311 | 40 | 18 | 2.3 | 87 |
| 2606306 | 2606364 | 58 | 27 | 2.2 | 87 |
| 4885700 | 4885762 | 62 | 30 | 2.1 | 87 |
| 211722 | 211783 | 61 | 30 | 2.1 | 87 |
| 6119905 | 6119966 | 61 | 30 | 2.1 | 87 |
| 3794168 | 3794214 | 46 | 24 | 2.0 | 87 |
| 6320863 | 6321154 | 291 | 159 | 1.8 | 87 |
| 3801695 | 3802040 | 345 | 36 | 9.7 | 86 |
| 3110517 | 3110630 | 113 | 15 | 7.6 | 86 |
| 2341558 | 2341624 | 66 | 9 | 7.4 | 86 |
| 3394562 | 3394696 | 134 | 18 | 7.2 | 86 |
| 4095600 | 4095664 | 64 | 9 | 6.9 | 86 |
| 2961544 | 2961687 | 143 | 30 | 4.8 | 86 |
| 2239053 | 2239188 | 135 | 30 | 4.5 | 86 |
| 895043 | 895176 | 133 | 42 | 3.2 | 86 |
| 2930275 | 2930338 | 63 | 20 | 3.2 | 86 |
| 5829918 | 5829981 | 63 | 20 | 3.2 | 86 |
| 2607638 | 2607770 | 132 | 45 | 3.0 | 86 |
| 4095574 | 4095686 | 112 | 39 | 2.9 | 86 |
| 4095574 | 4095685 | 111 | 39 | 2.9 | 86 |
| 2413183 | 2413260 | 77 | 27 | 2.9 | 86 |
| 2081982 | 2082016 | 34 | 12 | 2.9 | 86 |
| 4095423 | 4095457 | 34 | 12 | 2.9 | 86 |
| 5457155 | 5457189 | 34 | 12 | 2.9 | 86 |
| 177987 | 178020 | 33 | 12 | 2.8 | 86 |
| 4319455 | 4319488 | 33 | 12 | 2.8 | 86 |
| 2960245 | 2960324 | 79 | 30 | 2.7 | 86 |
| 4416329 | 4416376 | 47 | 18 | 2.7 | 86 |
| 953614 | 953824 | 210 | 80 | 2.6 | 86 |
| 2239031 | 2239188 | 157 | 60 | 2.6 | 86 |
| 556907 | 556985 | 78 | 30 | 2.6 | 86 |
| 2354773 | 2354819 | 46 | 18 | 2.6 | 86 |
| 4476190 | 4476235 | 45 | 18 | 2.6 | 86 |
| 1070476 | 1070551 | 75 | 30 | 2.5 | 86 |
| 2434088 | 2434161 | 73 | 30 | 2.5 | 86 |
| 3315055 | 3315127 | 72 | 30 | 2.4 | 86 |
| 321466 | 321528 | 62 | 26 | 2.4 | 86 |
| 554298 | 554365 | 67 | 30 | 2.3 | 86 |
| 554316 | 554383 | 67 | 30 | 2.3 | 86 |
| 2412444 | 2412484 | 40 | 18 | 2.3 | 86 |
| 3573225 | 3573265 | 40 | 18 | 2.3 | 86 |
| 6325519 | 6325559 | 40 | 18 | 2.3 | 86 |
| 1070024 | 1070090 | 66 | 30 | 2.2 | 86 |
| 213651 | 213715 | 64 | 30 | 2.2 | 86 |
| 1070044 | 1070108 | 64 | 30 | 2.2 | 86 |
| 3838731 | 3838795 | 64 | 30 | 2.2 | 86 |
| 1765056 | 1765108 | 52 | 24 | 2.2 | 86 |
| 3399124 | 3399176 | 52 | 24 | 2.2 | 86 |
| 7826 | 7865 | 39 | 18 | 2.2 | 86 |
| 2132693 | 2132732 | 39 | 18 | 2.2 | 86 |
| 546628 | 546666 | 38 | 18 | 2.2 | 86 |
| 5933063 | 5933101 | 38 | 18 | 2.2 | 86 |
| 551335 | 551372 | 37 | 18 | 2.2 | 86 |
| 5080601 | 5080669 | 68 | 33 | 2.1 | 86 |
| 2607789 | 2607844 | 55 | 27 | 2.1 | 86 |
| 5476338 | 5476381 | 43 | 21 | 2.1 | 86 |
| 2733385 | 2733424 | 39 | 18 | 2.1 | 86 |
| 2735148 | 2735187 | 39 | 18 | 2.1 | 86 |
| 4407124 | 4407229 | 105 | 54 | 2.0 | 86 |
| 4885458 | 4885515 | 57 | 30 | 2.0 | 86 |
| 4028205 | 4028248 | 43 | 22 | 2.0 | 86 |
| 1380662 | 1380704 | 42 | 21 | 2.0 | 86 |
| 2356921 | 2357006 | 85 | 45 | 1.9 | 86 |
| 1757560 | 1757617 | 57 | 30 | 1.9 | 86 |
| 950204 | 950249 | 45 | 24 | 1.9 | 86 |
| 3371113 | 3371431 | 318 | 40 | 8.0 | 85 |
| 2190073 | 2190121 | 48 | 9 | 5.4 | 85 |
| 1576401 | 1576443 | 42 | 9 | 4.8 | 85 |
| 313358 | 313419 | 61 | 15 | 4.1 | 85 |
| 2845442 | 2845479 | 37 | 10 | 3.8 | 85 |
| 5068907 | 5068961 | 54 | 15 | 3.6 | 85 |
| 5734996 | 5735055 | 59 | 18 | 3.3 | 85 |
| 48998 | 49035 | 37 | 12 | 3.3 | 85 |
| 2041539 | 2041596 | 57 | 18 | 3.2 | 85 |
| 1534879 | 1534939 | 60 | 21 | 2.9 | 85 |
| 4853104 | 4853146 | 42 | 15 | 2.9 | 85 |
| 2961868 | 2961950 | 82 | 30 | 2.8 | 85 |
| 636243 | 636348 | 105 | 39 | 2.7 | 85 |
| 5081285 | 5081361 | 76 | 30 | 2.6 | 85 |
| 3578723 | 3578784 | 61 | 24 | 2.6 | 85 |
| 3426572 | 3426625 | 53 | 22 | 2.6 | 85 |
| 2903121 | 2903165 | 44 | 18 | 2.5 | 85 |
| 3286 | 3328 | 42 | 16 | 2.5 | 85 |
| 3834196 | 3834267 | 71 | 30 | 2.4 | 85 |
| 3965811 | 3965882 | 71 | 30 | 2.4 | 85 |
| 2666464 | 2666510 | 46 | 20 | 2.4 | 85 |
| 3835596 | 3835665 | 69 | 30 | 2.3 | 85 |
| 546619 | 546679 | 60 | 27 | 2.3 | 85 |
| 546949 | 547009 | 60 | 27 | 2.3 | 85 |
| 3806495 | 3806542 | 47 | 21 | 2.3 | 85 |
| 4643616 | 4643651 | 35 | 15 | 2.3 | 85 |
| 1762943 | 1762981 | 38 | 18 | 2.2 | 85 |
| 2189786 | 2189982 | 196 | 93 | 2.1 | 85 |
| 546959 | 546996 | 37 | 18 | 2.1 | 85 |
| 3320347 | 3320492 | 145 | 72 | 2.0 | 85 |
| 3966014 | 3966132 | 118 | 60 | 2.0 | 85 |
| 546125 | 546178 | 53 | 27 | 2.0 | 85 |
| 226902 | 226942 | 40 | 21 | 2.0 | 85 |
| 1132617 | 1132657 | 40 | 21 | 2.0 | 85 |
| 1627549 | 1627589 | 40 | 21 | 2.0 | 85 |
| 4532905 | 4532945 | 40 | 21 | 2.0 | 85 |
| 1667554 | 1667593 | 39 | 21 | 2.0 | 85 |
| 5678782 | 5678821 | 39 | 21 | 2.0 | 85 |
| 2683015 | 2683052 | 37 | 20 | 2.0 | 85 |
| 6320734 | 6321282 | 548 | 288 | 1.9 | 85 |
| 2780136 | 2780562 | 426 | 231 | 1.9 | 85 |
| 466737 | 466858 | 121 | 63 | 1.9 | 85 |
| 559737 | 559794 | 57 | 30 | 1.9 | 85 |
| 4428895 | 4428951 | 56 | 30 | 1.9 | 85 |
| 1322991 | 1323030 | 39 | 21 | 1.9 | 85 |
| 3915677 | 3915715 | 38 | 21 | 1.9 | 85 |
| 2489755 | 2489928 | 173 | 96 | 1.8 | 85 |
| 2413199 | 2413275 | 76 | 9 | 8.6 | 84 |
| 2932658 | 2932718 | 60 | 9 | 6.8 | 84 |
| 2295256 | 2295323 | 67 | 11 | 6.3 | 84 |
| 3143113 | 3143200 | 87 | 15 | 5.8 | 84 |
| 2606811 | 2606858 | 47 | 9 | 5.3 | 84 |
| 1142993 | 1143034 | 41 | 9 | 4.7 | 84 |
| 2026092 | 2026133 | 41 | 9 | 4.7 | 84 |
| 2079891 | 2079932 | 41 | 9 | 4.7 | 84 |
| 1622058 | 1622139 | 81 | 20 | 4.1 | 84 |
| 4465777 | 4465810 | 33 | 9 | 3.8 | 84 |
| 466757 | 466823 | 66 | 18 | 3.7 | 84 |
| 559341 | 559447 | 106 | 30 | 3.6 | 84 |
| 3775061 | 3775136 | 75 | 21 | 3.6 | 84 |
| 3270818 | 3270934 | 116 | 36 | 3.3 | 84 |
| 559977 | 560075 | 98 | 30 | 3.3 | 84 |
| 558513 | 558558 | 45 | 15 | 3.1 | 84 |
| 5194111 | 5194267 | 156 | 54 | 3.0 | 84 |
| 4125933 | 4126005 | 72 | 24 | 3.0 | 84 |
| 1759179 | 1759257 | 78 | 27 | 2.9 | 84 |
| 5896284 | 5896341 | 57 | 20 | 2.9 | 84 |
| 17996 | 18327 | 331 | 120 | 2.8 | 84 |
| 2601964 | 2602013 | 49 | 18 | 2.8 | 84 |
| 4475562 | 4475611 | 49 | 18 | 2.8 | 84 |
| 2412428 | 2412476 | 48 | 18 | 2.7 | 84 |
| 5070183 | 5070223 | 40 | 15 | 2.7 | 84 |
| 2098113 | 2098152 | 39 | 15 | 2.7 | 84 |
| 2100906 | 2100945 | 39 | 15 | 2.7 | 84 |
| 2103726 | 2103765 | 39 | 15 | 2.7 | 84 |
| 1921536 | 1921644 | 108 | 40 | 2.6 | 84 |
| 6380417 | 6381127 | 710 | 285 | 2.5 | 84 |
| 2632919 | 2632992 | 73 | 30 | 2.4 | 84 |
| 5932997 | 5933039 | 42 | 18 | 2.4 | 84 |
| 3839443 | 3839510 | 67 | 30 | 2.3 | 84 |
| 1123083 | 1123141 | 58 | 27 | 2.2 | 84 |
| 2189899 | 2189944 | 45 | 21 | 2.2 | 84 |
| 4890500 | 4890651 | 151 | 72 | 2.1 | 84 |
| 4465998 | 4466108 | 110 | 54 | 2.1 | 84 |
| 2607690 | 2607784 | 94 | 45 | 2.1 | 84 |
| 5077139 | 5077200 | 61 | 30 | 2.1 | 84 |
| 5078423 | 5078484 | 61 | 30 | 2.1 | 84 |
| 3573311 | 3573368 | 57 | 27 | 2.1 | 84 |
| 2800926 | 2800971 | 45 | 21 | 2.1 | 84 |
| 5704543 | 5704588 | 45 | 21 | 2.1 | 84 |
| 1143062 | 1143106 | 44 | 21 | 2.1 | 84 |
| 5664979 | 5665068 | 89 | 45 | 2.0 | 84 |
| 548255 | 548328 | 73 | 36 | 2.0 | 84 |
| 671911 | 671963 | 52 | 27 | 2.0 | 84 |
| 2073284 | 2073332 | 48 | 24 | 2.0 | 84 |
| 547685 | 547736 | 51 | 27 | 1.9 | 84 |
| 2354768 | 2354819 | 51 | 27 | 1.9 | 84 |
| 1143806 | 1143845 | 39 | 21 | 1.9 | 84 |
| 2189285 | 2189433 | 148 | 15 | 9.9 | 83 |
| 3573295 | 3573361 | 66 | 9 | 7.4 | 83 |
| 4101671 | 4101712 | 41 | 6 | 7.0 | 83 |
| 3572905 | 3572966 | 61 | 9 | 6.9 | 83 |
| 2959734 | 2959890 | 156 | 30 | 5.2 | 83 |
| 3801695 | 3802037 | 342 | 72 | 4.8 | 83 |
| 4095180 | 4095347 | 167 | 36 | 4.7 | 83 |
| 1203086 | 1203156 | 70 | 15 | 4.7 | 83 |
| 3573383 | 3573422 | 39 | 9 | 4.4 | 83 |
| 2356731 | 2356769 | 38 | 9 | 4.3 | 83 |
| 5070541 | 5070665 | 124 | 30 | 4.2 | 83 |
| 561003 | 561060 | 57 | 15 | 3.9 | 83 |
| 562455 | 562512 | 57 | 15 | 3.9 | 83 |
| 3742080 | 3742131 | 51 | 15 | 3.8 | 83 |
| 562412 | 562516 | 104 | 30 | 3.5 | 83 |
| 2124165 | 2124369 | 204 | 60 | 3.4 | 83 |
| 3742092 | 3742132 | 40 | 12 | 3.4 | 83 |
| 554427 | 554526 | 99 | 30 | 3.3 | 83 |
| 5080063 | 5080162 | 99 | 30 | 3.3 | 83 |
| 2598873 | 2598930 | 57 | 18 | 3.2 | 83 |
| 2601705 | 2601762 | 57 | 18 | 3.2 | 83 |
| 2948461 | 2948540 | 79 | 27 | 3.0 | 83 |
| 5081419 | 5081462 | 43 | 15 | 2.9 | 83 |
| 2961879 | 2961962 | 83 | 30 | 2.8 | 83 |
| 2349505 | 2349553 | 48 | 18 | 2.7 | 83 |
| 2932651 | 2932719 | 68 | 27 | 2.6 | 83 |
| 4617596 | 4617642 | 46 | 18 | 2.6 | 83 |
| 6001010 | 6001055 | 45 | 18 | 2.6 | 83 |
| 1203085 | 1203156 | 71 | 30 | 2.4 | 83 |
| 1068016 | 1068081 | 65 | 30 | 2.2 | 83 |
| 3834068 | 3834132 | 64 | 30 | 2.2 | 83 |
| 2081941 | 2081997 | 56 | 27 | 2.1 | 83 |
| 3775061 | 3775143 | 82 | 42 | 2.0 | 83 |
| 3834502 | 3834561 | 59 | 30 | 2.0 | 83 |
| 16089 | 16147 | 58 | 30 | 2.0 | 83 |
| 19602 | 19660 | 58 | 30 | 2.0 | 83 |
| 173712 | 173770 | 58 | 29 | 2.0 | 83 |
| 1211467 | 1211514 | 47 | 24 | 2.0 | 83 |
| 3572289 | 3572538 | 249 | 132 | 1.9 | 83 |
| 3789619 | 3789757 | 138 | 75 | 1.9 | 83 |
| 3831711 | 3831824 | 113 | 60 | 1.9 | 83 |
| 5722684 | 5722770 | 86 | 45 | 1.9 | 83 |
| 211747 | 211804 | 57 | 30 | 1.9 | 83 |
| 3756617 | 3756849 | 232 | 126 | 1.8 | 83 |
| 2263403 | 2263607 | 204 | 20 | 10.2 | 82 |
| 2354768 | 2354819 | 51 | 9 | 5.8 | 82 |
| 6001956 | 6002004 | 48 | 9 | 5.4 | 82 |
| 3641176 | 3641280 | 104 | 20 | 5.3 | 82 |
| 5458109 | 5458195 | 86 | 18 | 4.8 | 82 |
| 4436470 | 4436520 | 50 | 11 | 4.6 | 82 |
| 213500 | 213563 | 63 | 15 | 4.3 | 82 |
| 1520620 | 1520751 | 131 | 33 | 4.1 | 82 |
| 555561 | 555678 | 117 | 30 | 3.9 | 82 |
| 1921548 | 1921615 | 67 | 20 | 3.4 | 82 |
| 3640932 | 3640999 | 67 | 20 | 3.4 | 82 |
| 2431708 | 2431845 | 137 | 42 | 3.3 | 82 |
| 1757837 | 1757885 | 48 | 15 | 3.3 | 82 |
| 2663795 | 2663889 | 94 | 30 | 3.2 | 82 |
| 6095287 | 6095347 | 60 | 20 | 3.0 | 82 |
| 16258 | 16608 | 350 | 120 | 2.9 | 82 |
| 19771 | 20112 | 341 | 120 | 2.9 | 82 |
| 3180462 | 3180564 | 102 | 36 | 2.9 | 82 |
| 6001928 | 6002004 | 76 | 27 | 2.9 | 82 |
| 4005467 | 4005523 | 56 | 18 | 2.9 | 82 |
| 3831395 | 3831437 | 42 | 15 | 2.9 | 82 |
| 3300369 | 3300453 | 84 | 30 | 2.8 | 82 |
| 953630 | 953787 | 157 | 60 | 2.6 | 82 |
| 3838327 | 3838404 | 77 | 30 | 2.6 | 82 |
| 3598472 | 3598518 | 46 | 18 | 2.6 | 82 |
| 5080963 | 5081037 | 74 | 30 | 2.5 | 82 |
| 1738997 | 1739063 | 66 | 27 | 2.5 | 82 |
| 3573295 | 3573361 | 66 | 27 | 2.5 | 82 |
| 1672210 | 1672254 | 44 | 18 | 2.5 | 82 |
| 3572213 | 3572256 | 43 | 18 | 2.5 | 82 |
| 1738249 | 1738454 | 205 | 87 | 2.4 | 82 |
| 5807619 | 5807705 | 86 | 36 | 2.4 | 82 |
| 190334 | 190398 | 64 | 27 | 2.4 | 82 |
| 4859334 | 4859403 | 69 | 30 | 2.3 | 82 |
| 3838490 | 3838557 | 67 | 30 | 2.3 | 82 |
| 3572666 | 3572726 | 60 | 27 | 2.3 | 82 |
| 3847234 | 3847283 | 49 | 22 | 2.3 | 82 |
| 18577 | 18706 | 129 | 60 | 2.2 | 82 |
| 627703 | 627754 | 51 | 24 | 2.2 | 82 |
| 1070296 | 1070424 | 128 | 60 | 2.1 | 82 |
| 562233 | 562358 | 125 | 60 | 2.1 | 82 |
| 559571 | 559634 | 63 | 30 | 2.1 | 82 |
| 6120054 | 6120117 | 63 | 30 | 2.1 | 82 |
| 5076218 | 5076279 | 61 | 30 | 2.1 | 82 |
| 895027 | 895082 | 55 | 27 | 2.1 | 82 |
| 4617621 | 4617705 | 84 | 42 | 2.0 | 82 |
| 5074715 | 5074773 | 58 | 30 | 2.0 | 82 |
| 53063 | 53108 | 45 | 24 | 2.0 | 82 |
| 3704990 | 3705059 | 69 | 36 | 1.9 | 82 |
| 5664980 | 5665049 | 69 | 36 | 1.9 | 82 |
| 5074593 | 5074650 | 57 | 30 | 1.9 | 82 |
| 555845 | 555901 | 56 | 30 | 1.9 | 82 |
| 8063 | 8228 | 165 | 90 | 1.8 | 82 |
| 2607792 | 2607844 | 52 | 9 | 5.9 | 81 |
| 3371113 | 3371400 | 287 | 60 | 4.8 | 81 |
| 3957911 | 3957949 | 38 | 9 | 4.3 | 81 |
| 3806492 | 3806557 | 65 | 18 | 3.7 | 81 |
| 2665019 | 2665130 | 111 | 30 | 3.6 | 81 |
| 4095618 | 4095727 | 109 | 30 | 3.6 | 81 |
| 5043073 | 5043118 | 45 | 12 | 3.6 | 81 |
| 2413063 | 2413123 | 60 | 18 | 3.4 | 81 |
| 5081063 | 5081161 | 98 | 30 | 3.3 | 81 |
| 3320347 | 3320453 | 106 | 36 | 3.0 | 81 |
| 2601351 | 2601404 | 53 | 18 | 3.0 | 81 |
| 2602767 | 2602820 | 53 | 18 | 3.0 | 81 |
| 4860206 | 4860293 | 87 | 30 | 2.9 | 81 |
| 3834243 | 3834326 | 83 | 30 | 2.8 | 81 |
| 3299794 | 3299876 | 82 | 30 | 2.8 | 81 |
| 1715305 | 1715369 | 64 | 23 | 2.8 | 81 |
| 2860768 | 2860826 | 58 | 21 | 2.8 | 81 |
| 2600194 | 2600244 | 50 | 18 | 2.8 | 81 |
| 2600902 | 2600952 | 50 | 18 | 2.8 | 81 |
| 2601256 | 2601306 | 50 | 18 | 2.8 | 81 |
| 2601610 | 2601660 | 50 | 18 | 2.8 | 81 |
| 2602672 | 2602722 | 50 | 18 | 2.8 | 81 |
| 2603026 | 2603076 | 50 | 18 | 2.8 | 81 |
| 2603380 | 2603430 | 50 | 18 | 2.8 | 81 |
| 2599840 | 2599889 | 49 | 18 | 2.8 | 81 |
| 2604443 | 2604492 | 49 | 17 | 2.8 | 81 |
| 6119875 | 6119915 | 40 | 15 | 2.7 | 81 |
| 2663546 | 2663585 | 39 | 15 | 2.7 | 81 |
| 2665482 | 2665521 | 39 | 15 | 2.7 | 81 |
| 2536635 | 2537169 | 534 | 207 | 2.6 | 81 |
| 211439 | 211516 | 77 | 30 | 2.6 | 81 |
| 3838701 | 3838778 | 77 | 30 | 2.6 | 81 |
| 6226049 | 6226097 | 48 | 18 | 2.6 | 81 |
| 3611660 | 3611701 | 41 | 15 | 2.5 | 81 |
| 8184 | 8374 | 190 | 81 | 2.4 | 81 |
| 5070522 | 5070665 | 143 | 60 | 2.4 | 81 |
| 3300055 | 3300125 | 70 | 30 | 2.4 | 81 |
| 2780103 | 2780172 | 69 | 30 | 2.4 | 81 |
| 1581206 | 1581260 | 54 | 24 | 2.3 | 81 |
| 3180526 | 3180678 | 152 | 69 | 2.2 | 81 |
| 556742 | 556871 | 129 | 60 | 2.2 | 81 |
| 2933181 | 2933247 | 66 | 30 | 2.2 | 81 |
| 558174 | 558239 | 65 | 30 | 2.2 | 81 |
| 2597496 | 2597819 | 323 | 156 | 2.1 | 81 |
| 5836922 | 5836982 | 60 | 29 | 2.1 | 81 |
| 82066 | 82114 | 48 | 23 | 2.1 | 81 |
| 6379353 | 6379492 | 139 | 72 | 2.0 | 81 |
| 559913 | 560000 | 87 | 45 | 2.0 | 81 |
| 3951893 | 3951946 | 53 | 27 | 2.0 | 81 |
| 2804893 | 2805059 | 166 | 87 | 1.9 | 81 |
| 545464 | 545532 | 68 | 36 | 1.9 | 81 |
| 4020059 | 4020128 | 69 | 9 | 7.8 | 80 |
| 4465993 | 4466122 | 129 | 18 | 7.2 | 80 |
| 6321038 | 6321932 | 894 | 129 | 6.9 | 80 |
| 203630 | 203690 | 60 | 9 | 6.8 | 80 |
| 3965937 | 3966112 | 175 | 30 | 5.9 | 80 |
| 6252347 | 6252407 | 60 | 10 | 5.8 | 80 |
| 4884942 | 4885111 | 169 | 30 | 5.7 | 80 |
| 953614 | 953823 | 209 | 40 | 5.3 | 80 |
| 554457 | 554526 | 69 | 15 | 4.7 | 80 |
| 4476200 | 4476241 | 41 | 9 | 4.7 | 80 |
| 562448 | 562511 | 63 | 15 | 4.3 | 80 |
| 2473388 | 2473451 | 63 | 15 | 4.3 | 80 |
| 2349515 | 2349553 | 38 | 9 | 4.3 | 80 |
| 1056464 | 1056501 | 37 | 9 | 4.2 | 80 |
| 5494594 | 5496923 | 2329 | 604 | 3.9 | 80 |
| 4428553 | 4428668 | 115 | 30 | 3.9 | 80 |
| 557945 | 558058 | 113 | 30 | 3.8 | 80 |
| 5795992 | 5796032 | 40 | 11 | 3.6 | 80 |
| 2606631 | 2606694 | 63 | 18 | 3.5 | 80 |
| 557976 | 558028 | 52 | 15 | 3.5 | 80 |
| 1203163 | 1203204 | 41 | 12 | 3.5 | 80 |
| 6321047 | 6321932 | 885 | 255 | 3.4 | 80 |
| 2607256 | 2607313 | 57 | 18 | 3.2 | 80 |
| 3773590 | 3773646 | 56 | 18 | 3.2 | 80 |
| 2665482 | 2665574 | 92 | 30 | 3.1 | 80 |
| 312770 | 312861 | 91 | 30 | 3.1 | 80 |
| 2598520 | 2598575 | 55 | 18 | 3.1 | 80 |
| 2599582 | 2599637 | 55 | 18 | 3.1 | 80 |
| 2600644 | 2600699 | 55 | 18 | 3.1 | 80 |
| 2600998 | 2601053 | 55 | 18 | 3.1 | 80 |
| 2603830 | 2603885 | 55 | 18 | 3.1 | 80 |
| 2604538 | 2604593 | 55 | 18 | 3.1 | 80 |
| 2960152 | 2960196 | 44 | 15 | 3.0 | 80 |
| 213465 | 213548 | 83 | 30 | 2.8 | 80 |
| 3832803 | 3832886 | 83 | 30 | 2.8 | 80 |
| 3837024 | 3837107 | 83 | 30 | 2.8 | 80 |
| 1763841 | 1763915 | 74 | 27 | 2.8 | 80 |
| 18051 | 18378 | 327 | 120 | 2.7 | 80 |
| 562793 | 562873 | 80 | 30 | 2.7 | 80 |
| 3741893 | 3741941 | 48 | 18 | 2.7 | 80 |
| 2081452 | 2081499 | 47 | 18 | 2.7 | 80 |
| 2239309 | 2239388 | 79 | 30 | 2.6 | 80 |
| 2757247 | 2757315 | 68 | 27 | 2.6 | 80 |
| 6001619 | 6001665 | 46 | 18 | 2.6 | 80 |
| 3573146 | 3573213 | 67 | 27 | 2.5 | 80 |
| 475472 | 475522 | 50 | 20 | 2.5 | 80 |
| 475726 | 475776 | 50 | 20 | 2.5 | 80 |
| 4548077 | 4548125 | 48 | 21 | 2.5 | 80 |
| 551347 | 551389 | 42 | 18 | 2.4 | 80 |
| 2356812 | 2356854 | 42 | 18 | 2.4 | 80 |
| 2606322 | 2606364 | 42 | 18 | 2.4 | 80 |
| 2734735 | 2734777 | 42 | 18 | 2.4 | 80 |
| 2960037 | 2960176 | 139 | 60 | 2.3 | 80 |
| 2597684 | 2597752 | 68 | 30 | 2.3 | 80 |
| 2732731 | 2732792 | 61 | 27 | 2.3 | 80 |
| 6004824 | 6004884 | 60 | 27 | 2.3 | 80 |
| 4315218 | 4315271 | 53 | 24 | 2.3 | 80 |
| 1758799 | 1758850 | 51 | 23 | 2.3 | 80 |
| 1715292 | 1715344 | 52 | 23 | 2.2 | 80 |
| 2684642 | 2684746 | 104 | 54 | 1.9 | 80 |
| 4248732 | 4250574 | 1842 | 1019 | 1.8 | 80 |
| 8095 | 8262 | 167 | 90 | 1.8 | 80 |
| 6201168 | 6201354 | 186 | 15 | 12.5 | 79 |
| 2732701 | 2732760 | 59 | 9 | 6.7 | 79 |
| 2597691 | 2597752 | 61 | 9 | 6.6 | 79 |
| 2732809 | 2732865 | 56 | 9 | 6.3 | 79 |
| 561311 | 561475 | 164 | 30 | 5.5 | 79 |
| 3270635 | 3270754 | 119 | 24 | 5.3 | 79 |
| 3573452 | 3573499 | 47 | 9 | 5.3 | 79 |
| 5070579 | 5070734 | 155 | 30 | 5.2 | 79 |
| 560870 | 560932 | 62 | 15 | 4.2 | 79 |
| 2734458 | 2734517 | 59 | 18 | 3.4 | 79 |
| 3838731 | 3838781 | 50 | 15 | 3.4 | 79 |
| 2239554 | 2239652 | 98 | 30 | 3.3 | 79 |
| 555696 | 555793 | 97 | 30 | 3.3 | 79 |
| 3835596 | 3835644 | 48 | 15 | 3.3 | 79 |
| 5995098 | 5995143 | 45 | 15 | 3.3 | 79 |
| 556616 | 556703 | 87 | 30 | 3.0 | 79 |
| 3706577 | 3706680 | 103 | 36 | 2.9 | 79 |
| 4428663 | 4428750 | 87 | 30 | 2.9 | 79 |
| 2732700 | 2732777 | 77 | 27 | 2.9 | 79 |
| 554196 | 554239 | 43 | 15 | 2.9 | 79 |
| 2958163 | 2958206 | 43 | 15 | 2.9 | 79 |
| 4859133 | 4859176 | 43 | 15 | 2.9 | 79 |
| 1520620 | 1520751 | 131 | 48 | 2.8 | 79 |
| 2960510 | 2960595 | 85 | 30 | 2.8 | 79 |
| 559752 | 559835 | 83 | 30 | 2.8 | 79 |
| 561102 | 561261 | 159 | 60 | 2.7 | 79 |
| 2341558 | 2341633 | 75 | 27 | 2.7 | 79 |
| 3572630 | 3572678 | 48 | 18 | 2.7 | 79 |
| 559871 | 560029 | 158 | 60 | 2.6 | 79 |
| 2098359 | 2098435 | 76 | 30 | 2.6 | 79 |
| 2101152 | 2101228 | 76 | 30 | 2.6 | 79 |
| 546577 | 547394 | 817 | 330 | 2.5 | 79 |
| 555150 | 555296 | 146 | 60 | 2.5 | 79 |
| 1069930 | 1070003 | 73 | 30 | 2.5 | 79 |
| 1069693 | 1069765 | 72 | 30 | 2.4 | 79 |
| 4860524 | 4860596 | 72 | 30 | 2.4 | 79 |
| 2795930 | 2796031 | 101 | 45 | 2.3 | 79 |
| 559692 | 559760 | 68 | 30 | 2.3 | 79 |
| 5458011 | 5458071 | 60 | 27 | 2.3 | 79 |
| 3573084 | 3573124 | 40 | 18 | 2.3 | 79 |
| 4465769 | 4465809 | 40 | 18 | 2.3 | 79 |
| 2958200 | 2958265 | 65 | 30 | 2.2 | 79 |
| 2948461 | 2948540 | 79 | 9 | 8.9 | 78 |
| 1542685 | 1542754 | 69 | 9 | 7.8 | 78 |
| 8195 | 8251 | 56 | 9 | 6.7 | 78 |
| 3270635 | 3270752 | 117 | 21 | 5.3 | 78 |
| 2341560 | 2341633 | 73 | 15 | 4.7 | 78 |
| 2097249 | 2097387 | 138 | 30 | 4.6 | 78 |
| 2100051 | 2100189 | 138 | 30 | 4.6 | 78 |
| 2102847 | 2102985 | 138 | 30 | 4.6 | 78 |
| 5917697 | 5917854 | 157 | 36 | 4.4 | 78 |
| 2860768 | 2860922 | 154 | 42 | 3.7 | 78 |
| 3832623 | 3832677 | 54 | 15 | 3.7 | 78 |
| 3836844 | 3836898 | 54 | 15 | 3.7 | 78 |
| 5841185 | 5841347 | 162 | 45 | 3.6 | 78 |
| 3835402 | 3835455 | 53 | 15 | 3.6 | 78 |
| 2663844 | 2663896 | 52 | 15 | 3.5 | 78 |
| 2433997 | 2434096 | 99 | 30 | 3.3 | 78 |
| 2599227 | 2599284 | 57 | 18 | 3.2 | 78 |
| 2599935 | 2599992 | 57 | 18 | 3.2 | 78 |
| 2602059 | 2602116 | 57 | 18 | 3.2 | 78 |
| 2602413 | 2602470 | 57 | 18 | 3.2 | 78 |
| 2603121 | 2603178 | 57 | 18 | 3.2 | 78 |
| 2603475 | 2603532 | 57 | 18 | 3.2 | 78 |
| 2604183 | 2604240 | 57 | 18 | 3.2 | 78 |
| 2605244 | 2605301 | 57 | 18 | 3.2 | 78 |
| 2605598 | 2605655 | 57 | 18 | 3.2 | 78 |
| 2605952 | 2606009 | 57 | 18 | 3.2 | 78 |
| 2600289 | 2600345 | 56 | 18 | 3.2 | 78 |
| 2604892 | 2604948 | 56 | 18 | 3.2 | 78 |
| 5080963 | 5081010 | 47 | 15 | 3.2 | 78 |
| 555597 | 555690 | 93 | 30 | 3.1 | 78 |
| 5664302 | 5664357 | 55 | 18 | 3.1 | 78 |
| 4884920 | 4885102 | 182 | 60 | 3.0 | 78 |
| 213652 | 213696 | 44 | 15 | 3.0 | 78 |
| 4475598 | 4475731 | 133 | 45 | 2.9 | 78 |
| 2665754 | 2665840 | 86 | 30 | 2.9 | 78 |
| 6380417 | 6381228 | 811 | 285 | 2.8 | 78 |
| 556697 | 556856 | 159 | 60 | 2.7 | 78 |
| 3757675 | 3757718 | 43 | 18 | 2.7 | 78 |
| 1993544 | 1993586 | 42 | 15 | 2.6 | 78 |
| 4891153 | 4891324 | 171 | 72 | 2.4 | 78 |
| 4891663 | 4891834 | 171 | 72 | 2.4 | 78 |
| 4893052 | 4893223 | 171 | 72 | 2.4 | 78 |
| 2124150 | 2124236 | 86 | 40 | 2.2 | 78 |
| 2079890 | 2079948 | 58 | 27 | 2.2 | 78 |
| 3808391 | 3808526 | 135 | 6 | 21.2 | 77 |
| 2124165 | 2124369 | 204 | 20 | 10.3 | 77 |
| 953630 | 953816 | 186 | 20 | 9.3 | 77 |
| 190267 | 190336 | 69 | 9 | 7.8 | 77 |
| 3572666 | 3572734 | 68 | 9 | 7.7 | 77 |
| 1765017 | 1765084 | 67 | 9 | 7.6 | 77 |
| 1764522 | 1764581 | 59 | 9 | 6.7 | 77 |
| 546125 | 546178 | 53 | 9 | 6.0 | 77 |
| 5722684 | 5722770 | 86 | 15 | 5.8 | 77 |
| 2356919 | 2357021 | 102 | 18 | 5.7 | 77 |
| 559916 | 560000 | 84 | 15 | 5.7 | 77 |
| 2957879 | 2958043 | 164 | 30 | 5.5 | 77 |
| 3573152 | 3573200 | 48 | 9 | 5.4 | 77 |
| 5664873 | 5664961 | 88 | 18 | 4.9 | 77 |
| 555150 | 555293 | 143 | 30 | 4.8 | 77 |
| 2431753 | 2431848 | 95 | 21 | 4.6 | 77 |
| 18577 | 18706 | 129 | 30 | 4.3 | 77 |
| 5722663 | 5722787 | 124 | 30 | 4.2 | 77 |
| 4892013 | 4893155 | 1142 | 294 | 3.9 | 77 |
| 5840192 | 5840354 | 162 | 45 | 3.6 | 77 |
| 3320347 | 3320471 | 124 | 36 | 3.5 | 77 |
| 4858867 | 4858966 | 99 | 30 | 3.3 | 77 |
| 2663396 | 2663493 | 97 | 30 | 3.3 | 77 |
| 551538 | 551597 | 59 | 18 | 3.3 | 77 |
| 5773752 | 5774152 | 400 | 126 | 3.2 | 77 |
| 2663167 | 2663254 | 87 | 30 | 2.9 | 77 |
| 4421690 | 4421732 | 42 | 16 | 2.7 | 77 |
| 3802921 | 3802966 | 45 | 18 | 2.5 | 77 |
| 2958510 | 2958578 | 68 | 30 | 2.3 | 77 |
| 549026 | 549088 | 62 | 27 | 2.3 | 77 |
| 3371126 | 3371431 | 305 | 20 | 15.3 | 76 |
| 4095583 | 4095706 | 123 | 9 | 12.8 | 76 |
| 2356919 | 2357021 | 102 | 9 | 11.4 | 76 |
| 3808394 | 3808541 | 147 | 15 | 10.7 | 76 |
| 953616 | 953823 | 207 | 20 | 10.4 | 76 |
| 203630 | 203690 | 60 | 6 | 10.2 | 76 |
| 2413063 | 2413140 | 77 | 9 | 8.7 | 76 |
| 1738306 | 1738367 | 61 | 9 | 6.9 | 76 |
| 5789229 | 5789288 | 59 | 9 | 6.7 | 76 |
| 551311 | 551363 | 52 | 9 | 5.9 | 76 |
| 556966 | 557216 | 250 | 45 | 5.6 | 76 |
| 561220 | 561470 | 250 | 45 | 5.6 | 76 |
| 3300369 | 3300452 | 83 | 15 | 5.6 | 76 |
| 545281 | 547062 | 1781 | 330 | 5.4 | 76 |
| 556697 | 556840 | 143 | 30 | 4.8 | 76 |
| 562554 | 562685 | 131 | 30 | 4.4 | 76 |
| 2606561 | 2606676 | 115 | 27 | 4.4 | 76 |
| 562229 | 562358 | 129 | 30 | 4.3 | 76 |
| 1921514 | 1921596 | 82 | 20 | 4.2 | 76 |
| 4095363 | 4095421 | 58 | 15 | 3.9 | 76 |
| 551406 | 551474 | 68 | 18 | 3.8 | 76 |
| 2932651 | 2932718 | 67 | 18 | 3.8 | 76 |
| 4890191 | 4892649 | 2458 | 663 | 3.7 | 76 |
| 6154270 | 6154325 | 55 | 15 | 3.7 | 76 |
| 2489617 | 2489779 | 162 | 45 | 3.6 | 76 |
| 5069154 | 5069257 | 103 | 30 | 3.5 | 76 |
| 555775 | 555824 | 49 | 15 | 3.4 | 76 |
| 4853312 | 4853411 | 99 | 30 | 3.3 | 76 |
| 5068922 | 5069015 | 93 | 30 | 3.1 | 76 |
| 1738997 | 1739079 | 82 | 27 | 3.1 | 76 |
| 5793890 | 5793979 | 89 | 30 | 3.0 | 76 |
| 1070292 | 1070423 | 131 | 45 | 2.9 | 76 |
| 5780583 | 5780634 | 51 | 18 | 2.9 | 76 |
| 1764434 | 1764510 | 76 | 27 | 2.8 | 76 |
| 4617547 | 4617684 | 137 | 54 | 2.6 | 76 |
| 3572648 | 3572734 | 86 | 36 | 2.4 | 76 |
| 2097597 | 2097660 | 63 | 27 | 2.4 | 76 |
| 2100399 | 2100462 | 63 | 27 | 2.4 | 76 |
| 2103195 | 2103258 | 63 | 27 | 2.4 | 76 |
| 548305 | 548348 | 43 | 18 | 2.4 | 76 |
| 1794671 | 1794713 | 42 | 18 | 2.4 | 76 |
| 2962510 | 2962593 | 83 | 36 | 2.3 | 76 |
| 1335360 | 1335428 | 68 | 32 | 2.2 | 76 |
| 3801725 | 3802035 | 310 | 18 | 17.3 | 75 |
| 4095149 | 4095347 | 198 | 12 | 16.6 | 75 |
| 3706574 | 3706689 | 115 | 9 | 12.9 | 75 |
| 3808376 | 3808569 | 193 | 15 | 11.9 | 75 |
| 2957879 | 2958026 | 147 | 15 | 9.9 | 75 |
| 556963 | 557219 | 256 | 30 | 8.6 | 75 |
| 5917695 | 5917847 | 152 | 18 | 8.5 | 75 |
| 5841274 | 5841345 | 71 | 9 | 8.0 | 75 |
| 561219 | 561455 | 236 | 30 | 7.9 | 75 |
| 3706761 | 3706837 | 76 | 9 | 7.9 | 75 |
| 4095567 | 4095715 | 148 | 21 | 7.5 | 75 |
| 2665019 | 2665130 | 111 | 15 | 7.3 | 75 |
| 3965997 | 3966100 | 103 | 15 | 6.9 | 75 |
| 2606565 | 2606682 | 117 | 18 | 6.7 | 75 |
| 561131 | 561231 | 100 | 15 | 6.7 | 75 |
| 2189783 | 2189982 | 199 | 33 | 6.4 | 75 |
| 562583 | 562668 | 85 | 15 | 5.7 | 75 |
| 18622 | 18706 | 84 | 15 | 5.7 | 75 |
| 4858388 | 4858550 | 162 | 30 | 5.4 | 75 |
| 4668862 | 4668953 | 91 | 18 | 5.1 | 75 |
| 5070104 | 5070176 | 72 | 15 | 4.9 | 75 |
| 2098719 | 2098786 | 67 | 15 | 4.6 | 75 |
| 2101512 | 2101579 | 67 | 15 | 4.6 | 75 |
| 2104332 | 2104399 | 67 | 15 | 4.6 | 75 |
| 5995420 | 5995473 | 53 | 12 | 4.6 | 75 |
| 2413027 | 2413140 | 113 | 27 | 4.2 | 75 |
| 3831285 | 3831407 | 122 | 30 | 4.1 | 75 |
| 3833068 | 3833129 | 61 | 15 | 4.1 | 75 |
| 3837289 | 3837350 | 61 | 15 | 4.1 | 75 |
| 2096752 | 2096872 | 120 | 30 | 4.0 | 75 |
| 2099554 | 2099674 | 120 | 30 | 4.0 | 75 |
| 2102350 | 2102470 | 120 | 30 | 4.0 | 75 |
| 562304 | 562362 | 58 | 15 | 3.9 | 75 |
| 3832563 | 3832785 | 222 | 60 | 3.7 | 75 |
| 3836784 | 3837006 | 222 | 60 | 3.7 | 75 |
| 627697 | 627740 | 43 | 12 | 3.7 | 75 |
| 562643 | 562857 | 214 | 60 | 3.6 | 75 |
| 2960508 | 2960559 | 51 | 15 | 3.5 | 75 |
| 2663859 | 2663909 | 50 | 15 | 3.4 | 75 |
| 2660085 | 2660132 | 47 | 15 | 3.2 | 75 |
| 2961879 | 2961924 | 45 | 15 | 3.1 | 75 |
| 5194290 | 5194366 | 76 | 27 | 2.9 | 75 |
| 5069214 | 5069257 | 43 | 15 | 2.9 | 75 |
| 2684784 | 2684826 | 42 | 15 | 2.9 | 75 |
| 3300241 | 3300323 | 82 | 30 | 2.8 | 75 |
| 2599132 | 2599181 | 49 | 18 | 2.8 | 75 |
| 2599486 | 2599535 | 49 | 18 | 2.8 | 75 |
| 2600548 | 2600597 | 49 | 18 | 2.8 | 75 |
| 2603734 | 2603783 | 49 | 18 | 2.8 | 75 |
| 2605151 | 2605198 | 47 | 17 | 2.8 | 75 |
| 3834345 | 3834427 | 82 | 30 | 2.7 | 75 |
| 3835641 | 3835721 | 80 | 30 | 2.7 | 75 |
| 4436464 | 4436520 | 56 | 22 | 2.6 | 75 |
| 5840262 | 5840311 | 49 | 18 | 2.5 | 75 |
| 2050444 | 2050530 | 86 | 9 | 9.7 | 74 |
| 2040727 | 2040795 | 68 | 9 | 7.3 | 74 |
| 562635 | 562847 | 212 | 30 | 7.1 | 74 |
| 1070296 | 1070400 | 104 | 15 | 7.0 | 74 |
| 1070236 | 1070424 | 188 | 30 | 6.3 | 74 |
| 3835467 | 3835546 | 79 | 15 | 5.3 | 74 |
| 2732701 | 2732792 | 91 | 18 | 5.1 | 74 |
| 4005467 | 4005552 | 85 | 15 | 4.9 | 74 |
| 6001927 | 6002004 | 77 | 18 | 4.3 | 74 |
| 18558 | 18662 | 104 | 30 | 3.5 | 74 |
| 1068317 | 1068419 | 102 | 30 | 3.4 | 74 |
| 3558615 | 3558684 | 69 | 21 | 3.4 | 74 |
| 5918126 | 5918186 | 60 | 18 | 3.4 | 74 |
| 3835762 | 3835805 | 43 | 15 | 2.9 | 74 |
| 4629343 | 4629686 | 343 | 126 | 2.8 | 74 |
| 2957879 | 2958043 | 164 | 60 | 2.7 | 74 |
| 560921 | 561080 | 159 | 60 | 2.7 | 74 |
| 2957909 | 2958026 | 117 | 45 | 2.6 | 74 |
| 2932787 | 2932853 | 66 | 27 | 2.5 | 74 |
| 4465993 | 4466122 | 129 | 9 | 14.4 | 73 |
| 3831670 | 3831823 | 153 | 15 | 10.3 | 73 |
| 5458107 | 5458195 | 88 | 9 | 9.9 | 73 |
| 3640974 | 3641074 | 100 | 11 | 9.5 | 73 |
| 1520616 | 1520751 | 135 | 15 | 8.7 | 73 |
| 1935989 | 1936275 | 286 | 38 | 7.5 | 73 |
| 6304562 | 6304623 | 61 | 9 | 7.0 | 73 |
| 5968790 | 5968857 | 67 | 9 | 6.9 | 73 |
| 3790584 | 3790645 | 61 | 9 | 6.9 | 73 |
| 3270818 | 3270934 | 116 | 18 | 6.7 | 73 |
| 556742 | 556840 | 98 | 15 | 6.6 | 73 |
| 14455 | 14515 | 60 | 9 | 6.4 | 73 |
| 5068922 | 5069015 | 93 | 15 | 6.3 | 73 |
| 6001004 | 6001055 | 51 | 9 | 5.8 | 73 |
| 556907 | 556991 | 84 | 15 | 5.7 | 73 |
| 3573328 | 3573422 | 94 | 18 | 5.3 | 73 |
| 4885004 | 4885083 | 79 | 15 | 5.3 | 73 |
| 560921 | 561064 | 143 | 30 | 4.8 | 73 |
| 1067576 | 1067704 | 128 | 30 | 4.3 | 73 |
| 22102 | 22166 | 64 | 15 | 4.3 | 73 |
| 1764160 | 1764232 | 72 | 18 | 4.1 | 73 |
| 3838144 | 3838249 | 105 | 30 | 3.5 | 73 |
| 2356919 | 2357014 | 95 | 27 | 3.5 | 73 |
| 6001778 | 6001838 | 60 | 18 | 3.4 | 73 |
| 1520663 | 1520720 | 57 | 18 | 3.4 | 73 |
| 22032 | 22130 | 98 | 30 | 3.3 | 73 |
| 2732815 | 2732897 | 82 | 27 | 3.1 | 73 |
| 4155097 | 4155143 | 46 | 15 | 3.1 | 73 |
| 6001096 | 6001166 | 70 | 27 | 2.6 | 73 |
| 4308481 | 4308538 | 57 | 24 | 2.5 | 73 |
| 561152 | 561455 | 303 | 15 | 20.3 | 72 |
| 556928 | 557201 | 273 | 15 | 18.3 | 72 |
| 4475353 | 4475488 | 135 | 9 | 14.8 | 72 |
| 562604 | 562817 | 213 | 15 | 14.3 | 72 |
| 3571708 | 3571828 | 120 | 9 | 13.4 | 72 |
| 3571568 | 3571631 | 63 | 6 | 10.7 | 72 |
| 1673194 | 1673290 | 96 | 9 | 10.4 | 72 |
| 4095627 | 4095737 | 110 | 12 | 10.3 | 72 |
| 1738997 | 1739073 | 76 | 9 | 8.6 | 72 |
| 2189817 | 2190010 | 193 | 33 | 6.2 | 72 |
| 556616 | 556708 | 92 | 15 | 6.2 | 72 |
| 3838327 | 3838404 | 77 | 15 | 5.2 | 72 |
| 3833073 | 3833136 | 63 | 15 | 4.3 | 72 |
| 3837294 | 3837357 | 63 | 15 | 4.3 | 72 |
| 5069140 | 5069254 | 114 | 30 | 3.9 | 72 |
| 6001560 | 6001662 | 102 | 27 | 3.8 | 72 |
| 2097247 | 2097302 | 55 | 15 | 3.7 | 72 |
| 2100049 | 2100104 | 55 | 15 | 3.7 | 72 |
| 2102845 | 2102900 | 55 | 15 | 3.7 | 72 |
| 3833942 | 3833997 | 55 | 15 | 3.7 | 72 |
| 559356 | 559410 | 54 | 15 | 3.7 | 72 |
| 3371551 | 3371618 | 67 | 17 | 3.5 | 72 |
| 2804985 | 2805049 | 64 | 18 | 3.4 | 72 |
| 559347 | 559397 | 50 | 15 | 3.4 | 72 |
| 313021 | 313119 | 98 | 30 | 3.3 | 72 |
| 4892036 | 4892496 | 460 | 150 | 3.1 | 72 |
| 4892108 | 4892568 | 460 | 150 | 3.1 | 72 |
| 22276 | 22344 | 68 | 30 | 2.3 | 72 |
| 2607503 | 2607766 | 263 | 9 | 29.0 | 71 |
| 2189879 | 2189949 | 70 | 6 | 12.8 | 71 |
| 4475605 | 4475688 | 83 | 9 | 9.0 | 71 |
| 3832563 | 3832783 | 220 | 30 | 7.4 | 71 |
| 3836784 | 3837004 | 220 | 30 | 7.4 | 71 |
| 559951 | 560060 | 109 | 15 | 7.4 | 71 |
| 2189801 | 2190010 | 209 | 27 | 7.1 | 71 |
| 5070522 | 5070621 | 99 | 15 | 6.7 | 71 |
| 3802354 | 3802492 | 138 | 21 | 6.3 | 71 |
| 1249680 | 1249764 | 84 | 15 | 6.2 | 71 |
| 1947543 | 1947604 | 61 | 12 | 5.7 | 71 |
| 1764478 | 1764572 | 94 | 18 | 5.3 | 71 |
| 555597 | 555675 | 78 | 15 | 5.3 | 71 |
| 4475598 | 4475731 | 133 | 27 | 4.9 | 71 |
| 2757119 | 2757186 | 67 | 15 | 4.9 | 71 |
| 313450 | 313521 | 71 | 15 | 4.8 | 71 |
| 2239699 | 2239769 | 70 | 15 | 4.7 | 71 |
| 5664975 | 5665046 | 71 | 18 | 4.0 | 71 |
| 5423134 | 5423201 | 67 | 18 | 3.8 | 71 |
| 2903150 | 2903224 | 74 | 21 | 3.7 | 71 |
| 5665234 | 5665290 | 56 | 18 | 3.2 | 71 |
| 2932559 | 2932612 | 53 | 18 | 2.9 | 71 |
| 2607485 | 2607755 | 270 | 18 | 15.4 | 70 |
| 2607485 | 2607764 | 279 | 27 | 10.3 | 70 |
| 555150 | 555301 | 151 | 15 | 10.1 | 70 |
| 1947535 | 1947626 | 91 | 9 | 9.6 | 70 |
| 2961544 | 2961682 | 138 | 15 | 9.3 | 70 |
| 3808361 | 3808544 | 183 | 24 | 7.7 | 70 |
| 2804980 | 2805049 | 69 | 9 | 7.4 | 70 |
| 3801261 | 3801509 | 248 | 36 | 6.9 | 70 |
| 2096761 | 2096846 | 85 | 15 | 5.8 | 70 |
| 2099563 | 2099648 | 85 | 15 | 5.8 | 70 |
| 2102359 | 2102444 | 85 | 15 | 5.8 | 70 |
| 2355201 | 2355302 | 101 | 18 | 5.5 | 70 |
| 2079725 | 2079813 | 88 | 18 | 5.2 | 70 |
| 2860774 | 2860880 | 106 | 21 | 5.1 | 70 |
| 555591 | 555667 | 76 | 15 | 5.1 | 70 |
| 4858448 | 4858521 | 73 | 15 | 4.9 | 70 |
| 4548121 | 4548203 | 82 | 21 | 4.2 | 70 |
| 554315 | 554369 | 54 | 15 | 3.7 | 70 |
| 2597656 | 2597792 | 136 | 39 | 3.5 | 70 |
| 2757071 | 2757145 | 74 | 24 | 3.4 | 70 |
| 2536635 | 2536942 | 307 | 105 | 2.9 | 70 |
| 5969245 | 5969295 | 50 | 18 | 2.8 | 70 |
| 2796124 | 2796266 | 142 | 9 | 15.9 | 69 |
| 2355202 | 2355302 | 100 | 9 | 10.9 | 69 |
| 2239206 | 2239357 | 151 | 15 | 10.1 | 69 |
| 4891874 | 4893168 | 1294 | 144 | 8.8 | 69 |
| 2663138 | 2663259 | 121 | 15 | 8.1 | 69 |
| 2960073 | 2960191 | 118 | 15 | 7.9 | 69 |
| 4475345 | 4475478 | 133 | 18 | 7.3 | 69 |
| 2959782 | 2959890 | 108 | 15 | 7.3 | 69 |
| 4892108 | 4893168 | 1060 | 144 | 7.2 | 69 |
| 2431697 | 2431845 | 148 | 21 | 7.1 | 69 |
| 4890866 | 4891269 | 403 | 72 | 5.5 | 69 |
| 2732815 | 2732892 | 77 | 18 | 4.3 | 69 |
| 2932762 | 2932860 | 98 | 27 | 3.7 | 69 |
| 2795896 | 2795957 | 61 | 18 | 3.4 | 69 |
| 215621 | 215706 | 85 | 27 | 3.3 | 69 |
| 5559724 | 5559773 | 49 | 15 | 3.3 | 69 |
| 544987 | 545197 | 210 | 9 | 23.8 | 68 |
| 2081942 | 2082097 | 155 | 9 | 17.3 | 68 |
| 3833974 | 3834140 | 166 | 15 | 11.1 | 68 |
| 5917849 | 5917978 | 129 | 18 | 7.2 | 68 |
| 1738997 | 1739111 | 114 | 18 | 6.2 | 68 |
| 2903150 | 2903271 | 121 | 21 | 6.1 | 68 |
| 4467517 | 4467621 | 104 | 18 | 5.7 | 68 |
| 4892026 | 4892434 | 408 | 72 | 5.6 | 68 |
| 3706751 | 3706895 | 144 | 30 | 4.6 | 68 |
| 2434028 | 2434096 | 68 | 15 | 4.6 | 68 |
| 8274 | 8333 | 59 | 18 | 3.5 | 68 |
| 5075972 | 5076023 | 51 | 16 | 3.4 | 68 |
| 5077247 | 5077298 | 51 | 16 | 3.4 | 68 |
| 5078531 | 5078582 | 51 | 16 | 3.4 | 68 |
| 2757067 | 2757208 | 141 | 9 | 17.4 | 67 |
| 2489572 | 2489694 | 122 | 9 | 13.3 | 67 |
| 6001078 | 6001166 | 88 | 9 | 9.9 | 67 |
| 3831299 | 3831407 | 108 | 15 | 7.3 | 67 |
| 1068132 | 1068341 | 209 | 30 | 7.0 | 67 |
| 2663396 | 2663493 | 97 | 15 | 6.5 | 67 |
| 2939599 | 2939696 | 97 | 18 | 5.4 | 67 |
| 2939619 | 2939716 | 97 | 18 | 5.4 | 67 |
| 3801405 | 3801507 | 102 | 24 | 4.3 | 67 |
| 4892482 | 4892595 | 113 | 36 | 3.2 | 67 |
| 4892518 | 4892631 | 113 | 36 | 3.2 | 67 |
| 3572294 | 3572592 | 298 | 9 | 31.9 | 66 |
| 2780099 | 2780217 | 118 | 9 | 12.7 | 66 |
| 4548110 | 4548235 | 125 | 15 | 7.8 | 66 |
| 4858754 | 4858919 | 165 | 30 | 5.3 | 66 |
| 2795934 | 2796003 | 69 | 18 | 3.9 | 66 |
| 3394887 | 3394959 | 72 | 20 | 3.6 | 66 |
| 695133 | 695190 | 57 | 18 | 3.2 | 66 |
| 2097291 | 2097408 | 117 | 15 | 7.9 | 65 |
| 2100093 | 2100210 | 117 | 15 | 7.9 | 65 |
| 2102889 | 2103006 | 117 | 15 | 7.9 | 65 |
| 1764450 | 1764591 | 141 | 27 | 5.3 | 65 |
| 2684640 | 2684720 | 80 | 18 | 4.5 | 65 |
| 2804592 | 2804660 | 68 | 18 | 3.8 | 65 |
| 5046945 | 5047019 | 74 | 24 | 3.3 | 65 |
| 137209 | 137493 | 284 | 31 | 9.2 | 64 |
| 3180467 | 3180563 | 96 | 18 | 5.4 | 64 |
| 3802345 | 3802413 | 68 | 24 | 3.0 | 64 |
| 2239101 | 2239179 | 78 | 15 | 5.3 | 63 |
| 4728954 | 4729039 | 85 | 26 | 3.2 | 63 |
| 215635 | 215767 | 132 | 27 | 5.1 | 62 |

Grey color indicates VNTR loci selected for further analysis.

^a^ Flanking sequence length [bp] in *M. kansasii* ATCC 12478.

**SUPPLEMENTARY TABLE 5.** Comparison of repeat copy number assessed with Sanger sequencing and gel sizing for VNTR 19, 20 and 24.

| Isolate | VNTR19 | | VNTR20 | | VNTR24 | |
| --- | --- | --- | --- | --- | --- | --- |
|  | Sequencing | Gel | Sequencing | Gel | Sequencing | Gel |
| TYPE I: ATTC12478 | 10.7 | 11 | 12.8 | 13 | 8.7 | 9 |
| TYPE II: (H2)48 | 12 | 12 | ND | 12 | ND | 3 |
| TYPE II: NLA0010011128 | 12 | 12 | ND | 12 | 3 | 3 |
| TYPE III: 174.15 | NP | NP^c^ | ND | 8 | ND | 9 |
| TYPE IV: 241.15 | 6 (+3)^a^ | 6 | NP | NP | 8.7 | 9 |
| TYPE V: 1010001454 | 8 (+2)^a^ | 7 | ND | 2 | ND | 9 |
| TYPE VI: NLA001001166 | ND^b^ | 10 | ND | 0 | 8.7 | 9 |

^a^ Number of identified truncated VNTR copies is given in brackets;

^b^ Sequence not determined;

^c^ No PCR product upon electrophoresis.
